# Platform-based manufacturing of tumor-infiltrating lymphocytes derived from different solid tumor types

**DOI:** 10.3389/fimmu.2026.1854761

**Published:** 2026-10-05

**Authors:** Justin Lievense, Liselotte Tas, Maaike van Zon, Kari-Anne Legué-van Duinen, Rosa van Amerongen, Sander Kouwen, Rosalie van Riessen, Femke Sluiter, Jeltsje Hagg, Madelon Badoux, Iris Manders, Eva Vedder, Erica Sellink, Tom Seijkens, Koen Hartemink, Marcus Rijken, Oscar Brouwer, Suzanne Castenmiller, Marten Hansen, Monika Wolkers, John Haanen, Jos Beijnen, Cynthia Nijenhuis, Inge Jedema, Bastiaan Nuijen

**Affiliations:** 1 BioTherapeutics Unit, The Netherlands Cancer Institute-Antoni van Leeuwenhoek, Amsterdam, Netherlands; 2 Division of Molecular Oncology and Immunology, The Netherlands Cancer Institute-Antoni van Leeuwenhoek, Amsterdam, Netherlands; 3 Department of Pharmacy & Pharmacology, The Netherlands Cancer Institute-Antoni van Leeuwenhoek, Amsterdam, Netherlands; 4 Department of Research Laboratory for Cell Therapy, Sanquin Blood Supply Foundation, Amsterdam, Netherlands; 5 Department of Research T-cell differentiation lab, Sanquin Blood Supply Foundation, Amsterdam, Netherlands; 6 Division of Medical Oncology, The Netherlands Cancer Institute-Antoni van Leeuwenhoek, Amsterdam, Netherlands; 7 Department of Surgery, The Netherlands Cancer Institute-Antoni van Leeuwenhoek, Amsterdam, Netherlands; 8 Department of Pulmonary Disease, Leiden University Medical Center, Leiden, Netherlands; 9 Department of Gynaecologic Oncology, The Netherlands Cancer Institute-Antoni van Leeuwenhoek, Amsterdam, Netherlands; 10 Julius Centre for Health Science and Primary Care, University Medical Center Utrecht, Utrecht, Netherlands; 11 Department of Urology, The Netherlands Cancer Institute-Antoni van Leeuwenhoek, Amsterdam, Netherlands; 12 Oncode Institute, Utrecht, Netherlands; 13 Landsteiner Laboratory, Amsterdam Medical Center, Amsterdam, Netherlands; 14 Department of Medical Oncology, Leiden University Medical Center, Leiden, Netherlands; 15 The Melanoma Clinic, Centre Hospitalier Universitaire Vaudois, Lausanne, Switzerland; 16 Department of Pharmaceutical Sciences, Utrecht University, Utrecht, Netherlands

**Keywords:** cellular therapy, good manufacturing practice, platform-based manufacturing, solid tumors, tumor-infiltrating lymphocytes

## Abstract

**Background:**

In adoptive cell therapy with tumor-infiltrating lymphocytes (TILs), TILs are isolated and expanded *ex vivo* from an autologous tumor lesion. TIL therapy, comprising preparative lymphodepleting chemotherapy, TIL infusion, and post-infusion supportive interleukin-2 (IL-2) administration, has demonstrated efficacy in patients with metastatic melanoma. Interest in extending TIL therapy to other solid tumors is growing, and a tumor-agnostic manufacturing platform would allow first-in-human trials to be launched rapidly in these indications.

**Objective:**

This study investigated whether a manufacturing process and control strategy developed for metastatic melanoma-derived TILs could be adapted into a tumor-agnostic platform for the generation of TILs from different solid tumor types, referred to as NTU101.

**Methods:**

The feasibility of generating TILs from endometrial carcinoma (ENDC, n=9), non-small cell lung cancer (NSCLC, n=9), and penile cancer (PENC, n=10) lesions was assessed during process development (PD) runs using predefined quality criteria. Throughout PD runs, flow cytometry was used to characterize cells. When available, autologous tumor digest was used to assess the tumor-reactive potential of TILs. Based on PD experience, adjusted quality criteria were applied to manufacture one full-scale GMP NTU101-drug product (DP) per tumor type.

**Results:**

Overall, 89% (n=25/28) of PD NTU101-DPs manufactured met the predefined quality criteria, containing at least 0.5×10^9 and 90% viable CD45+CD3+ cells, less than 1.0% of cellular impurities (viable CD45- and CD19+ cells), and a potency of more than 1.0×10^3 pg/mL IFN-γ secretion upon CD3/CD28-polymer stimulation. Across the tumor types, PD NTU101-DPs had comparable T-cell subset and differentiation state compositions. *In vitro* tumor-reactive potential was detected in 82% of tested NTU101-DPs (ENDC: 71%, n=5/7; NSCLC: 80%, n= 4/5; and PENC: 100%, n=5/5), although the level of reactivity will need to be confirmed with a more in-depth assay. One GMP NTU101-DP was then manufactured from each tumor type, and all three met NTU101’s quality criteria.

**Conclusion:**

The NTU101 platform produced comparable NTU101-DPs from three solid tumor types beyond melanoma, with *in vitro* tumor-reactive potential for some batches. This platform can be applied in TIL therapy clinical trials to generate ENDC-, NSCLC-, and PENC-derived TILs, and possibly other solid tumor types.

## 1 Introduction

In adoptive cell therapy with tumor-infiltrating lymphocytes (TILs), TILs are isolated and expanded *ex vivo* from a patient’s autologous tumor lesion. TIL therapy involves a preparative lymphodepleting chemotherapy regimen, followed by TIL infusion and subsequent supportive interleukin-2 (IL-2) administration. This approach has demonstrated efficacy, especially in patients with metastatic melanoma (1–4). The ability of TIL therapy to induce durable complete responses, even in heavily pretreated melanoma patients (2, 5), has sparked interest in its therapeutic potential for patients with other solid tumors.

Endometrial carcinoma (ENDC), non-small cell lung cancer (NSCLC), and penile cancer (PENC) are solid tumors with a significant unmet clinical need in the advanced or recurrent disease setting. Despite advancements in available systemic therapies, including immune checkpoint inhibitors or targeted treatments, many patients do not achieve durable responses and have limited options after progression (6–12). These patients may benefit from novel therapies capable of inducing durable anti-tumor responses, such as TIL therapy.

ENDC, NSCLC, and PENC tumors share biological characteristics that support TIL therapy as a feasible treatment option, including improved therapeutic outcomes in more densely CD45+CD3+ infiltrated tumors (13–15). Tumor-infiltrating, tumor-reactive CD45+CD3+ cells can recognize neoantigens arising from patient-specific mutations, tumor-associated antigens (TAAs), or viral antigens in virus-driven tumors, which account for approximately 25–61% of PENC cases (16, 17). A high tumor mutational burden (TMB) is present in most (smoking-induced) NSCLC tumors and in a subset of PENC and ENDC tumors, often due to mismatch repair (MMR) deficiency and/or microsatellite instability-high (MSI-H) status. This elevated TMB promotes neoantigen expression and supports the presence of tumor-reactive CD45+CD3+ cells (18–21). In metastatic melanoma, these tumor-reactive cells have been shown to expand and persist during the TIL manufacturing processes (22, 23). Given the diversity of antigens that can be recognized, expansion of tumor-infiltrating CD45+CD3+ cells from resected ENDC, NSCLC, and PENC tumors is likely to yield tumor-reactive CD45+CD3+ cells as well.

Previous studies have demonstrated the feasibility of generating TILs from ENDC (24), NSCLC (25, 26), and PENC lesions (27), where NSCLC-derived TILs have also demonstrated efficacy in first-in-human clinical trials (28). To generate these TILs, some studies developed tumor-type-specific manufacturing processes through the addition of agonistic αCD3 and/or αCD137 antibodies or the use of αCD3/CD28 nanobeads to provide activation/costimulatory signals during TIL manufacturing (26, 27, 29). Different cell culture platforms have also been used to generate TILs, ranging from gas-permeable flasks (30) and rocking-motion perfusion bioreactors (31, 32), to closed automated systems (33). The resulting generally tumor-type-specific processes effectively generated TILs. However, using a uniform process to generate TILs within a tumor-agnostic platform offers the important advantage of accelerating the initiation of first-in-human trials treating patients with different tumor types, if the platform is sufficiently robust.

The Netherlands Cancer Institute and Sanquin Blood Supply have extensive experience in producing TM001, a TIL product used to treat patients with metastatic melanoma with demonstrated efficacy (1). TM001 products robustly met quality criteria during TIL manufacturing, despite variability in the melanoma tumor starting material. This manufacturing robustness suggests that a tumor-agnostic manufacturing platform based on TM001’s process, using starting material from different tumor types, could also yield high-quality TIL products.

This study assessed whether TM001’s good manufacturing practice (GMP)-compliant manufacturing process and control strategy could be used to develop a tumor-agnostic manufacturing platform (hereafter, NTU101 platform). This platform used a uniform manufacturing process, quality control (QC), and release strategy that could be applied across heterogeneous tumor-derived starting materials from different tumor types, which ideally would result in NTU101 products with comparable quality attributes. The quality of PD NTU101 batches was initially evaluated using the predefined quality criteria for TM001 manufacturing. Then, the successful outgrowth of tumor-reactive CD45+CD3+ cells was confirmed to evaluate NTU101’s potential efficacy. If successful, the NTU101 platform could enable the production of high-quality TIL products from ENDC, NSCLC, and PENC lesions, thereby facilitating clinical evaluation in these solid tumor types and potentially broadening access to TIL therapy for patients with other solid tumor types.

## 2 Materials and methods

### 2.1 Tumor lesion procurement

Tumor material was collected from patients who underwent standard surgical resection of primary or metastatic ENDC, NSCLC, or PENC lesions between 2022 and 2024, irrespective of prior treatment history. All patients provided consent for the use of residual resected tumor material for research purposes. This study was reviewed and approved by the local Institutional Review Board (IRBm21-137). Tumors were eligible for inclusion in the manufacturing process if they were ≥2cm on a diagnostic CT scan. This criterion was based on a previous study that reported that TIL manufacturing was more likely to achieve CD45+CD3+ outgrowth and tumor-reactive CD45+CD3+ cells when using excised metastatic melanoma lesions with a CT-scan diameter of ≥2cm (34). Excised tumors were sent to pathology to confirm the presence of malignant cells in the tumor and for molecular classification, and residual tumor material was used for NTU101 manufacturing at the processing facility.

### 2.2 Manufacturing of NTU101

NTU101 batches were generated using the manufacturing processes based on TM001 manufacturing (1), as overviewed in **Figure 1A**, with two modifications. First, whilst the TM001 process used different cell culture media types for different steps, the NTU101 process used the same cell culture medium throughout all processing steps. Second, whilst both processes isolated viable cells from excised tumor lesions using enzymatic digestion, the NTU101 process applied a different digestion protocol. This included changing the digestion reagents, increasing the digestion temperature from room temperature (RT) to 37 °C, and shortening the digestion duration from four hours to 45 minutes. The subsequent steps were identical, where CD45+CD3+ cells were grown out of the tumor in the proliferation step and then expanded to clinically relevant doses in the rapid expansion phase (REP). In both TM001 and NTU101, viable CD45+CD3+ cells are the active substance in the drug substance (DS). At the end of the REP, the DS was harvested and formulated into the NTU101-drug product (DP) in IV-bags, a fresh medicinal product consisting of a viable CD45+CD3+ cell dispersion in 0.9% NaCl with 2.5% human serum albumin (HSA). TM001 DP has always been administered fresh to the patient within 6 hours after harvest and formulation, which will be the aim for NTU101 as well.

**Figure 1:**
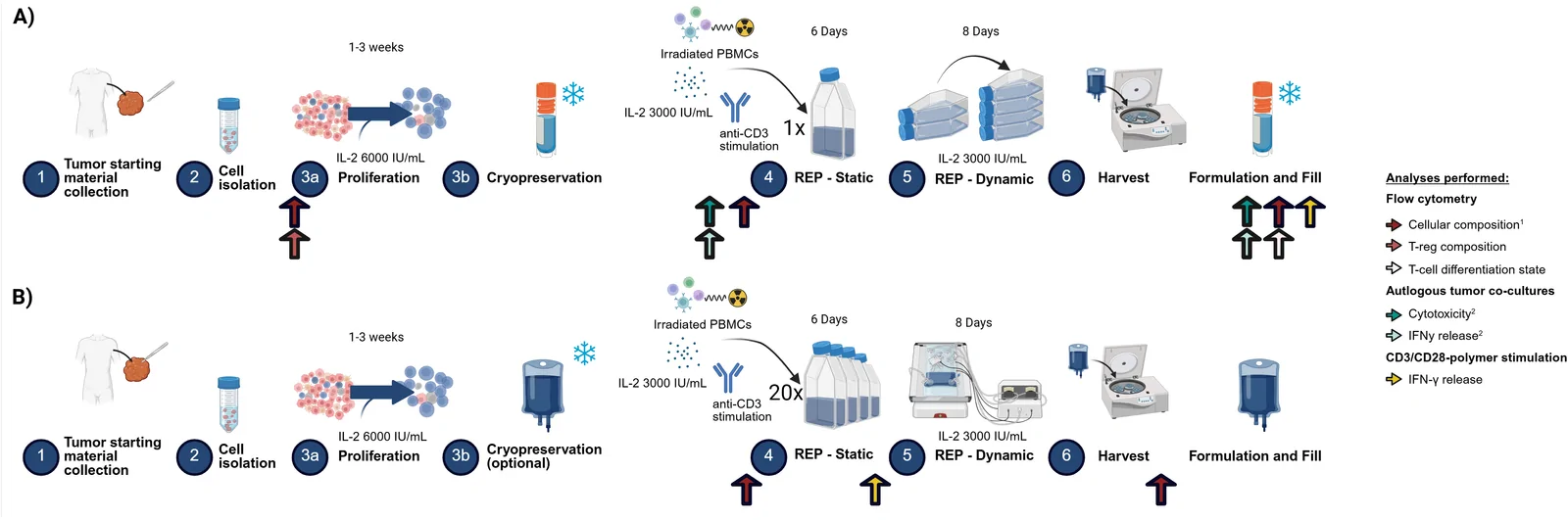
NTU101 manufacturing step overview and sampling points for the analyses performed for **(A)** process development NTU101 and **(B)** good manufacturing practice NTU101. PBMC, peripheral blood mononuclear cells; IL-2, interleukin-2. 1 = also used to determine in-process control action limits and release tests; 2 = characterized when sufficient residual autologous tumor digest was available for co-culture experiments and when the tumor digest contained >5% viable CD45- cells (including tumor cells).

Feasibility of using the NTU101 platform for tumor-agnostic TIL manufacturing was initially evaluated during process development (PD), using a process designed to be comparable to the GMP process (**Figure 1B**). For instance, NTU101 manufactured during PD (hereafter, PD NTU101) used the same GMP-compliant reagents at the same concentrations as GMP NTU101 batches. The main difference was the manufacturing scale. The REP in the PD NTU101 process was performed at 1/20^th^ the scale of GMP NTU101 manufacturing, initiated in a single T-175 flask (static), and used T-flasks throughout the 14-day culture period. In contrast, for GMP NTU101 manufacturing, the REP was split into a static phase (day 1-6) initiated in 20 T-175 flasks, and a dynamic phase (day 6-14) where CD45+CD3+ cells are cultured in a bioreactor (Xuri W25, Cytiva). Lastly, the CD45+CD3+ cell activation conditions at the start of the REP were the same in PD and GMP manufacturing, but with a more variable ratio of irradiated “feeder” peripheral mononuclear blood cells (PBMCs) to CD45+CD3+ cells. Multiple feeder cell lots were used throughout the study, consisting of pooled PBMCs from buffy coats of 6 or 15 donors for PD and GMP manufacturing, respectively. For GMP manufacturing, feeder cell lots were released for use as a raw material based on PBMC immune subset composition and individual donor viral testing specifications. Pooling PBMCs of multiple donors in one feeder cell lot averaged raw material attributes and mitigated potential donor-to-donor variability in supporting REP expansion. Similarly, multiple heat-inactivated human serum (HS) lots were used throughout the study. Pooling of 8 donors in one HS lot was performed to mitigate potential donor-to-donor variability. All HS donors were selected according to EU Directive 2002/98/EC and 2004/33/EC.

#### 2.2.1 Process development NTU101 batch manufacturing

Excised tumor lesions were trimmed, meaning that necrotic and non-tumor tissue (i.e., blood vessels and fat tissue) were removed. Hereafter, the three dimensions of the remaining material were measured to calculate the tumor volume. The remaining material was mechanically fragmented and enzymatically digested in 20/80 T-cell medium (20% AIM-V/80% RPMI, Thermo Fisher Scientific, custom-made) with 12.5 μg/ml DNAse (Pulmozyme, Roche), 0.17 IU/mL Neutral Protease (Nordmark), and 1 IU/mL Collagenase NB (Nordmark) at 37 °C for 45 minutes. Viable mononuclear cells were then isolated using density gradient separation (Ficoll-Paque, Cytiva or Lymphoprep, Serumwerk Bernburg).

The proliferation step was initiated in 24-well tissue culture plate(s), seeding a target of 1×10^6 isolated cells per well in complete medium (CM), consisting of 20/80 T-cell medium with 10% heat-inactivated HS (Sanquin Blood Supply), and 50 µg/mL gentamicin (Centrafarm), supplemented with 6,000 IU/mL IL-2 (Proleukin, Iovance). Residual isolated cells from the digested tumor were cryopreserved in CryoStor CS10 (Stemcell Technologies) in cryovials using freezer containers (Coolcell, Corning) for flow cytometry and autologous tumor co-culture analysis, as described in **Figure 1A**.

During the proliferation step, every 3–4 days, cells from one representative well were resuspended and counted using a brightfield microscope and hemocytometer. Processing decisions were based on the concentration of viable non-tumor cells, distinguished from viable tumor cells based on their size and morphology. When the viable non-tumor cell concentration was <1×10^6, half the CM medium supplemented with 6,000 IU/mL IL-2 was replaced in all the wells of the 24-well plate. When the viable non-tumor cell concentration was ≥1×10^6, all wells were split 1:1 using CM medium supplemented with 6,000 IU/mL IL-2. This continued for 1–3 weeks until IPC2–5 criteria were met (**Table 1**), ending the proliferation step. End-of-proliferation NTU101, referred to as the NTU101 intermediate product, was resuspended in 0.9% NaCl (Fresenius Kabi), supplemented with 2% human serum albumin (HSA), and mixed 1:1 with CryoStor CS10 before cryopreservation in cryovials for further processing, co-cultures, and flow cytometry (**Figure 1A**).

**Table 1:** Process development (PD) NTU101 and good manufacturing practice (GMP) NTU101 manufacturing quality criteria, including in-process controls (IPCs), release tests (RT), and sampling times.

Manufacturing step	Sampling time	Parameter	Analytical method	Quality criteria: GMP NTU101 (PD NTU101)
*NTU101-DS*				
***In-process controls (IPCs)***				***Action limits^1^***
Step 1: Tumor starting material collection &Step 2: Cell isolation	*None*			
Step 3a: Proliferation	End Day -1	**IPC1**: Sterility^2^	Ph. Eur. 2.6.27 (growth-based method)	No growth (NA)
		**IPC2:** Viable CD45+CD3+ cells (%)^3^	Flow cytometry (cell composition panel)	≥10.0%
	End	**IPC3:** Viable CD45+CD3+ cell number	Calculation	≥10×10^6 cells
		**IPC4:** Viable non-tumor cell fold expansion	Calculation	>2-fold
		**IPC5:** % viable tumor cells	Calculation	<10%
Step 3b: Cryopreservation (optional^2^)	Start	**IPC6:** Viable CD45+CD3+ cell number^2^	Calculation	≥80×10^6 cells (NA)
Thawing of cells (if cryopreserved)	*None*			
Step 4: REP –Static (Day 1 - 6)	Start	I**PC7:** Viable CD45+CD3+ cell number	Calculation	≥10×10^6 cells (≥0.5×10^6 cells)
		**IPC8:** Ratio of viable feeders:viable CD45+CD3+ cells	Calculation	200:1 ±10% (100-200:1)
	Day 6	**IPC9:** Viable cell fold expansion	Calculation	>10-fold
		**IPC10:** Feeder cell - proliferation	Calculation	<1.0-fold
		**IPC11:** Viable cell number^2^	Ph. Eur. 2.7.29	≥0.75×10^9 cells (NA)
**Release Tests (RTs)**				**Acceptance criteria’s**
Step 5: REP –Dynamic (Day 6 - 14)	Start (Day 6)	**RT1:** Potency^4^	ELISA	≥0.5×10^3 pg/mL IFN-γ^5^ (≥1.0×10^3 pg/mL IFN-γ)
		**RT2:** Sterility^2^	Ph.Eur. 2.6.27 (growth-based method)	No growth (NA)
	End (Day 14)	**RT3:** Viable CD45+CD3+ cells (%)	Flow cytometry (cell composition panel)	≥90.0%
		**RT4:** Viable CD45- cells (%)	Flow cytometry (cell composition panel)	<1.0%
		**RT5:** Viable CD19+ cells (%)	Flow cytometry (cell composition panel)	<1.0%
Step 6: Harvest	Supernatant fraction: End	**RT6:** Mycoplasma^1^	ddPCR	Not detected (NA)
*NTU101-DP*				
NTU101 Formulation and fill	NTU101-DP	**RT7:** Appearance^2^	Visual inspection	Drug product in the infusion bag. The bag is clean and intact, and there is no sign of leaks. Cell suspension white to off-white turbid liquid. No foreign particles or clumps. (NA)
		**RT8:** Viability (%)	Ph. Eur. 2.7.29	≥70%
		**RT9:** Viable CD45+CD3+ cell number	Calculation	≥10×10^9 cells (0.5×10^9 cells)
		**RT10:** Endotoxins^2^	Ph. Eur. 2.6.14 (chromogenic kinetic method)	≤1.2 EU/mL (NA)
		**RT11:** Sterility^2^	Ph. Eur. 2.6.27 (growth-based method)	No growth (NA)

^1^Meeting IPC action limits was not a requirement for NTU101 batch release, in contrast to RT acceptance criteria. When NTU101 batches were outside of IPC action limit ranges, in-process manufacturing conditions were more likely to result in out-of-specification NTU101 RTs, as the conditions deviated from normal manufacturing conditions. Therefore, by evaluating IPC action limits throughout manufacturing, timely risk assessments and potentially corrective and/or preventative actions could be performed. ^2^only for GMP NTU101 manufacturing, ^3^sampled on the day before ending (End Day -1) the proliferation step for GMP NTU101 batches, but from the cryopreserved and thawed intermediate products in PD, ^4^sampled on day 6 of the Rapid expansion for GMP NTU101 batches, but from the formulated product for the PD NTU101 batches. ^5^lowered from ≥1.0×10^3 pg/mL, based on data from PD NTU101 batches. ddPCR, digital droplet PCR;DS, drug substance;DP, Drug product;REP, Rapid expansion phase.

For initiation of the REP, the cryopreserved cells from the NTU101 intermediate product were thawed and cultured overnight at 37 °C in CM supplemented with 6,000 IU/mL IL-2 to recover from cryopreservation. The following day, these cells were used to initiate the REP in a T-175 culture flask at a seeding density of approximately 1×10^6 viable CD45+CD3+ cells in CM. CD45+CD3+ cells were activated by adding irradiated feeder cells in a 100-200:1 ratio (viable feeder cell: viable CD45+CD3+ cells), 30 ng/mL agonistic anti-CD3 antibody (OKT-3 clone, Miltenyi), and 3,000 IU/mL IL-2 to the flask, after which the flask was incubated in an upright position at 37 °C for 6 days. At day 6, two-thirds of the medium was replaced with fresh CM supplemented with 3,000 IU/mL IL-2, and IPC9 and 10 (**Table 1**) were evaluated. On day 7-8, REP cultures were split 1:1 using CM supplemented with 3,000 IU/mL IL-2, and again every 2–3 days until day 14. After the REP, NTU101-DS was harvested and resuspended in 0.9% NaCl, supplemented with 2% HSA, and 1:1 mixed with CryoStor CS10 for cryopreservation of PD NTU101-DP in cryovials for functional assays and characterization, which included RT assays (**Figure 1A**).

#### 2.2.2 GMP NTU101 manufacturing

For GMP NTU101 batches, the enzymatic digestion, cell isolation, and proliferation steps were performed as described above (**Figure 1B**), but in an EU GMP grade B cleanroom. Open handling steps were performed in a grade A microbial safety cabinet with a grade B background.

The static REP was initiated in 20 T-175 culture flasks containing 20×10^6 viable CD45+CD3+ cells, rather than 1×10^6 viable CD45+CD3+ cells (**Figure 1**), with feeder cells added in a 200:1 ± 10% viable feeder cell: viable CD45+CD3+ cell ratio. On day 6 of the REP, the dynamic phase was initiated by transferring all expanded cells to a Xuri W25 Bioreactor (closed-system), and the cells were automatically cultured in 4700 mL. During days 6–14 of the REP, an increasing volume of 20/80 T-cell medium supplemented with 3,000 IU/mL IL-2 (but without HS and gentamicin) was perfused into the bioreactor. After the REP, NTU101-DS was harvested, washed, and formulated (0.9% NaCl, 2.5% HSA) in IV-bags (Compoflex, Fresenius Kabi) to obtain the GMP NTU101-DP, a fresh cell dispersion for infusion.

### 2.3 Analytical methods and quality control

PD NTU101 cell cultures were characterized throughout manufacturing as described in **Figure 1A**, with the analytical methods detailed in the sections below. The analyses were retrospectively performed on cryopreserved samples, sampled as described in **Figure 1A**, with the exception of viable cell counting, which was performed on fresh samples. GMP NTU101 cell cultures were characterized as described in **Figure 1B**.

Some of the characterization data were used to evaluate the quality of PD NTU101 batches. The quality of NTU101 batches was initially evaluated using the predefined quality criteria for TM001 manufacturing, including release test (RT) acceptance criteria and in-process control (IPC) action limits, detailed in **Table 1**. RT acceptance criteria were used to identify out-of-specification NTU101 batches, which were of insufficient quality. PD and GMP NTU101 manufacturing was considered successful when the predefined RT acceptance criteria were met. In contrast to RT acceptance criteria, meeting IPC action limits was not a requirement for NTU101 batch release. When NTU101 batches were outside of IPC action limit ranges, in-process manufacturing conditions were more likely to result in out-of-specification NTU101 RTs, as the conditions deviated from normal manufacturing conditions for TM001. Therefore, by evaluating IPC action limits throughout manufacturing, timely risk assessments and potentially corrective and/or preventative actions could be performed during the manufacturing process runtime.

For GMP NTU101, the performed analysis (IPCs and RTs as described in **Table 1**, with justifications provided for the quality criteria in **Supplementary Table 1**) used fresh samples. One exception was RT6 (mycoplasma; **Table 1**), which was determined retrospectively from a cryopreserved sample.

#### 2.3.1 Viable cell counting

Viable cell numbers were determined from fresh cell cultures using a hemocytometer-based method, with non-viable cell exclusion with Trypan blue (Gibco or Sigma-Aldrich) (Ph. Eur. 2.7.29). In the proliferation step, tumor cells were differentiated from non-tumor cells using a brightfield microscope and a hemocytometer, based on cell size and morphology. Viable CD45+CD3+ cell numbers were determined by multiplying the viable cell number by the percentage of viable CD45+CD3+ cells in the sample determined by flow cytometry.

#### 2.3.2 Flow cytometry

Cell composition (including T-cell subsets), regulatory T cells (Tregs), and T-cell differentiation state were characterized using flow cytometry with three antibody panels (detailed in **Supplementary Tables 2**–**4**), at the manufacturing steps described in **Figure 1**. When used to characterize tumor digests, a target of 200.000 cells was stained per replicate, with a minimum of 20.000 cells based on cell availability. For the NTU101 intermediate product/DP, 200.000 cells were stained. For all panels, cells were stained with an IR-dye live/dead marker (Thermo Fisher Scientific). For the Treg and T-cell differentiation state panels, cells were permeabilized and fixed using a FoxP3/transcription factor staining buffer set (Invitrogen).

Data acquisition was performed on a spectral flow cytometer (NL-CLC, Cytek) maintained in a GMP environment, with a daily quality control procedure where gains were normalized using QC beads (SpectroFlo QC beads, Cytek). Spectral unmixing was performed using the automated live unmixing function in SpectroFlo software (v2.2.0.4; Cytek). Single-stain reference controls were generated using capture beads for all antibodies, and an unstained cell sample was used as a negative reference. Data were processed using FlowJo software (v10.0, BD), using fixed gating strategies for the cell composition of tumor digest and NTU101 intermediate product/DPs (**Supplementary Figures 1**, **2**, respectively). A fixed gating strategy was also used to characterize Tregs (**Supplementary Figure 3**). For T-cell differentiation state, a standardized gating strategy (**Supplementary Figure 4**) was normalized on a PBMC control sample per analytical run.

To enable the evaluation of quality criteria of GMP NTU101 batches (**Table 1**), the cell composition panel was validated in accordance with ICH Q2 guidelines and Ph. Eur. 2.7.24 and included two assay controls, a reference TIL product sample and a PBMC sample, with specifications measured in each analytical run. This assay determined the purity of viable CD45+CD3+ cells of NTU101 intermediate product (IPC2) and GMP NTU101-DS (RT3). The assay also quantified two cellular impurities, CD45- (e.g., including potential tumor cells, RT4), and CD19+ (B-) cells (RT5) in GMP NTU101-DS to exclude potential outgrowth of autologous tumor cells and/or Epstein Barr virus (EBV) transformed autologous B cells during the manufacturing process, which pose a safety risk upon adoptive transfer of the NTU-101 DP (35, 36).

#### 2.3.3 IFN-γ release upon CD3/CD28-polymer stimulation

To determine the potency of PD and GMP NTU101-DPs, IFN-γ release was quantified using ELISA after T-cell stimulation with a CD28/CD3-polymer (TransAct, Miltenyi). The analytical method was validated in accordance with ICH Q2 guidelines. The validated quantifiable range for IFN-γ was 62.5–12,500 pg/mL in non-activated samples and 625–50,000 pg/mL in activated samples, with standard curves required to meet accuracy and R²>0.95 acceptance criteria. The potency sample was sampled at day 6 of the REP (RT 1, **Table 1**) to enable fresh batch release of GMP NTU101. Fresh (GMP NTU101, sampled as described in **Table 1**) or thawed (PD NTU101-DP) samples were seeded in 24-well plates in CM medium supplemented with 100 IU/mL IL-2, and incubated at 37 °C for around 24 hours. After recovery, 10×10^3 viable cells were incubated with TransAct (1:100 dilution) or without (NC) in a 96-well plate for 16–18 hours in triplicate per sample and condition. The plate was centrifuged, and the supernatant was transferred to a new 96-well plate for IFN-γ quantification.

Secreted IFN-γ was quantified using reagents from, and a protocol based on, a human IFN-γ ELISA kit (Diaclone); this included the IFN-γ standards, antibody pair (capture and detection antibody), and horseradish peroxidase (HRP)-streptavidin, prepared as described in the kit’s protocol. In brief, a 96-well-ELISA plate (Thermo Fisher Scientific) was coated using the capture antibody (1:100 diluted in PBS), and incubated at 2-8 °C overnight. After incubation, the coated plate was washed with washing buffer (0.05% Tween 20 (Merck) in PBS) three times consecutively, and then incubated with blocking buffer (50 mg/mL BSA in 1x PBS) on a plate shaker at RT for two hours. Then, the plate was washed, after which the calibration range, quality controls, supernatant samples, and the detection antibody were applied and incubated on a plate shaker at RT for two hours. The plate was washed, after which HRP-streptavidin was added and then incubated on a plate shaker at RT for 30 minutes. For each analytical run, the 3,3’,5,5’-tetramethylbenzidine (TMB; Sigma-Aldrich) substrate solution was prepared in-house. The plate was washed, and the TMB substrate solution was incubated for 11 minutes before adding a sulphuric acid stop solution (Merck). Absorbance was measured at 450nm on a plate reader (BioTek 800TSNB, Agilent) and processed using Gen5 Secure software.

#### 2.3.4 Safety – sterility, bacterial endotoxins, and mycoplasma

During GMP NTU101 manufacturing, quality criteria for sterility, bacterial endotoxins, and mycoplasma were determined. Here, sterility was determined throughout manufacturing (IPC1, RT2, RT11; **Table 1**) using a (semi-) automated growth-based method (BacT/ALERT 3D, BioMérieux) in accordance with Ph. Eur. 2.6.27. The level of bacterial endotoxins (RT10; **Table 1**) was determined using a chromogenic kinetic method (Endosafe Nexgen-PTS, Charles River Laboratories) in accordance with Ph. Eur. 2.6.14. The absence of mycoplasma (RT6; **Table 1**) was confirmed using digital droplet PCR (ddPCR) in accordance with Ph. Eur 2.6.7. and 2.6.21, using a Droplet Reader (QXDx 200, Bio-Rad), according to a protocol from a ddPCR Mycoplasma detection kit (Vericheck, Bio-Rad).

#### 2.3.5 *In vitro* tumor-reactivity

*In vitro* tumor-reactivity was characterized for PD NTU101 batches when sufficient residual autologous tumor digest was available for co-culture experiments and when the tumor digest contained >5% viable CD45- cells (including tumor cells), determined with the cell composition flow cytometry panel. When possible, tumor-reactivity was evaluated for the NTU101 intermediate product and NTU101 DP.

##### 2.3.5.1 Cytotoxicity upon co-culture of CD45+CD3+ in NTU101 intermediate products and NTU101 DPs with autologous tumor digest

Cytotoxicity of *ex vivo* expanded CD45+CD3+ cells in NTU101 intermediate product and NTU101-DP (effector: E) was evaluated by co-culturing with autologous tumor digest cells (target: T) at various effector-to-target ratios (E:T; 0:1 (Negative control; NC), 0.3:1, 1:1, 5:1, 10:1) or conditions.

Cryopreserved NTU101 intermediate product and NTU101-DP samples were thawed, and 1×10^6 cells per well in 2 mL CM were plated in 24-well plates to recover for around 24 hours. After recovery, the tumor digest was thawed and incubated with 10% (v/v) Pulmozyme in 20/80 medium at 37 °C for 20 minutes. The cells in the tumor digest were then stained using cell trace violet (CTV; CellTrace, Invitrogen) according to the manufacturer’s instructions. All cells were resuspended in fresh CM before co-culture. For each E:T ratio condition, 25×10^3 viable CTV-stained tumor digest cells/well were transferred to 96-well plates in duplicate, whilst the NC condition was plated in quintuplicate as an internal control. Then effector cells were added at the required E:T ratios, and the wells were supplemented with CM to a total volume of 200 μL. The plates were incubated at 37 °C, and all cells in the co-culture were harvested after 16–20 hours.

Cytotoxicity was then quantified using flow cytometry. Cells from the co-culture and NC conditions were stained with an APC-labeled CD45 anti-human antibody (555485, BD Bioscience) and an IR-dye live-dead marker (LIVE/DEAD, Invitrogen). A fixed number of cell counting beads (CountBright, Thermo Fischer Scientific) was added per well, and the number of tumor cells in the tumor digest (CTV-stained CD45- cells) was quantified using a spectral flow cytometer (NL-CLC, Cytek), with the gating strategy described in **Supplementary Figure 5**, and calculated per condition as follows:


$${\text{Tumor cells}}_{total}=\left(\frac{{\text{cell counting beads}}_{total}}{{\text{cell counting beads}}_{measured}}\right)\times {\text{Tumor cells}}_{measured}\;$$

The % cytotoxicity per E:T condition was then calculated as follows:


$$\%{\text{cytotoxicity}}_{E:T}=1-\frac{{\text{Tumor cells}}_{total,E:T}}{{\text{Tumor cells}}_{total,NC}}$$

To be considered evaluable, the NC condition was required to have >100 mean CTV-stained CD45- events. The percentage reduction in CTV-stained CD45- cells at the various E:T ratios compared to the NC condition was reported as the percentage cytotoxicity. When the CD45- cell number was not lower for the E:T ratio conditions than the NC condition, cytotoxicity was considered zero percent.

##### 2.3.5.2 IFN-γ secretion upon co-culture of CD45+CD3+ cells in NTU101 intermediate products and NTU101 DPs with autologous tumor digest

IFN-γ secretion of CD45+CD3+ cells in NTU101 intermediate product and NTU101-DP upon co-culture with autologous tumor digest was quantified using ELISA. NTU101 intermediate product, NTU101-DP, and tumor digest were thawed as described for the cytotoxicity assay. 25×10^3 cells from NTU101 (R) were co-cultured in a 1:5 responder-to-stimulator (R:S) ratio with tumor digest cells (S) in CM, as well as the same number of NTU101 cells and tumor digest cells alone as NCs, in 96-well plates at 37 °C. After 16–20 hours of co-culture, the plates were centrifuged, and the supernatant was harvested from each condition and stored at -20 °C for further analysis. Secreted IFN-γ was quantified using the same method and reagents as described above for the potency assay. The fold change was calculated by dividing the highest IFN-γ concentration in NTU101 intermediate product/NTU101 DP alone or tumor digest alone by the concentration measured in the co-culture.

##### 2.3.5.3 Definition of tumor-reactivity

PD NTU101 intermediate products and DPs were considered tumor-reactive if the co-cultures in the cytotoxicity assay met the following two conditions: 1) increased cytotoxicity with higher E:T ratios (0.3:1 vs 10:1), demonstrating a dose-response relationship, 2) the mean cytotoxicity in the 10:1 condition exceeded the NC mean by more than the %coefficient of variance (CV) of the NC replicates. Alternatively, IFN-γ concentrations in the 1:5 R:S condition had to be more than 1.5-fold of the NC condition.

#### 2.3.6 Statistical analysis

Data analysis was performed in Python (v3.11.9). The normality of numerical data was assessed using Shapiro-Wilk tests for each tumor type. The homogeneity of variance between the tumor types was evaluated using Levene’s test. Normally distributed data with homogeneity of variance were compared using ANOVA. If one of these conditions was not met, data were compared using a Kruskal-Wallis test. Following a significant ANOVA or Kruskal-Wallis result (p ≤ 0.05), pairwise comparisons were performed using Tukey’s honest significant difference (HSD) or Dunn’s test, respectively, to identify specific group differences. P-values from Dunn’s test were adjusted for multiple comparisons using the Bonferroni correction. Non-parametric Spearman rank-order correlations (Spearman’s ρ) were utilized for all bivariate analyses evaluating associations.

## 3 Results

### 3.1 Patient and tumor starting material characteristics

Excised tumors from 28 patients undergoing routine surgical resection of ENDC (n=9), NSCLC (n=9), or PENC (n=10) were used as starting materials in PD NTU101 runs to evaluate the feasibility of tumor-agnostic TIL manufacturing. In addition, one GMP NTU101-DP was manufactured per tumor type. **Table 2** summarizes the patient and starting-material characteristics of the processed materials. Starting materials included both primary and metastatic tumors from patients with various stages of disease, treatment history, and diverse biological characteristics. These characteristics included both MMR-deficient and MMR-proficient ENDC tumors, HPV-positive and negative PENC tumors, and PD-L1-positive and negative NSCLC tumors.

**Table 2:** Patient and tumor starting material characteristics.

Characteristic	All	Endometrial carcinoma (ENDC)	Non-small cell lung cancer (NSCLC)	Penile cancer (PENC)
	N=31	N=10^1^	N=10^1^	N=11^1^
Age, years, median (range)	71 (40 – 88)	71 (50 – 77)	68 (40 – 81)	74 (52 – 88)
Sex, n male (% male of tumor type)	16 (51.6%)	0 (0)	5 (50.0)	11 (100.0)^1^
Tumor type, n (% of total)				
Endometroid adenocarcinoma	3 (9.7)	3 (30.0)	NA	NA
Endometrial serous carcinoma	5 (16.1)^2^	5 (50.0)^1,2^	NA	NA
Endometrial carcinosarcoma	1 (3.2)	1 (10.0)	NA	NA
Endometrial adenosarcoma	1 (3.2)	1 (10.0)	NA	NA
Lung adenocarcinoma	7 (22.5)	NA	7 (70.0)	NA
Lung Large cell neuroendocrine carcinoma	1 (3.2)	NA	1 (10.0)	NA
Lung squamous cell carcinoma	1 (3.2)	NA	1 (10.0)	NA
Non-small cell lung cancer of unknown subtype	1 (3.2)	NA	1 (10.0)^1^	NA
Penile squamous cell carcinoma	10 (31.3)	NA	NA	11 (100.0)^1^
Tumor type-specific characteristic, n (% of tumor type)				
MMR deficient	1 (3.2)	1 (10.0)	ND	ND
MMR proficient	8 (25.8)	8 (80.0)^1^	ND	ND
MSS	5 (16.1)	5 (50.0)^1,4^	ND	ND
Smoker	7 (22.6)	ND	7 (70%)^1^	ND
HPV positive	2 (6.5)	ND	ND	2 (18.1)
Excised lesion characteristics, n (% of tumor type)				
Primary	18 (58.1)	8 (80.0)^1^	4 (40.0)	6 (54.5)^1^
Metastasis	11 (35.5)	2 (20.0)	6 (60.0)^1^	3 (27.2)
Lymph node	3 (9.7)	0 (0.0)	0 (0.0)	3 (27.2)
Adrenal	3 (9.7)	0 (0.0)	3 (30.0)	0 (0.0)
Other	5 (16.1)	2 (20.0)	3 (30.0)^1^	0 (0.0)
Primary and Metastasis (mixed)	2 (6.5)	0 (0.0)	0 (0.0)	2 (18.1)^3^
Pre-treatment history, n (% of tumor type)				
Treatment Naive	25 (80.6)	10 (100.0)^1^	4 (40.0)	11 (100.0)^1^
Chemotherapy and Immunotherapy	6 (19.4)	0 (0.0)	6 (60.0)^1^	0 (0.0)
Extent of disease, n (% of tumor type)				
Local	17 (54.8)	7 (70.0)^1^	4 (40.0)	6 (54.5)^1^
Locoregional	7 (22.6)	1 (10.0)	1 (10.0)	5 (45.5)
Distant metastasis	7 (22.6)	2 (20.0)	5 (50.0)^1^	0 (0.0)

^1^Includes one GMP NTU101 batch, ^2^of which one was also classified as an endometrial carcinosarcoma, ^3^in these cases, a primary tumor and a lymph node metastasis were used to manufacture one PD NTU101 DP, ^4^all MSS ENDC tumors were also MMR proficient, ND, no data; NA, not applicable; MMR, mismatch repair; MSS, microsatellite stable; HPV, human papillomavirus.

### 3.2 Process development of NTU101

The excised tumor starting material was pre-processed, mechanically fragmented, and enzymatically digested into a single-cell suspension. The cell compositions in tumor digests were characterized using flow cytometry. The cell composition of the tumor digest showed substantial intertumoral variability (**Figure 2A**). Most tumor digests contained immune cells, mainly subsets of CD45+CD3+ cells (T cells) and CD45+CD14+ cells (monocytes), and to a lesser extent, CD45+CD3-CD56+ cells (Natural killer cells; NK cells), CD45+SSChighCD16+ cells (granulocytes), and CD45+CD19+ cells (B cells) (**Supplementary Table 5**). The percentage of CD45- cells (including tumor cells) of viable cells ranged from 0.2%–81% (**Supplementary Table 5**), with percentages that were significantly higher in ENDC tumor digests compared with PENC (38.8% vs 3.2%, respectively; p<0.001), while NSCLC was in between (median, 14.3%; **Figure 2B**).

**Figure 2:**
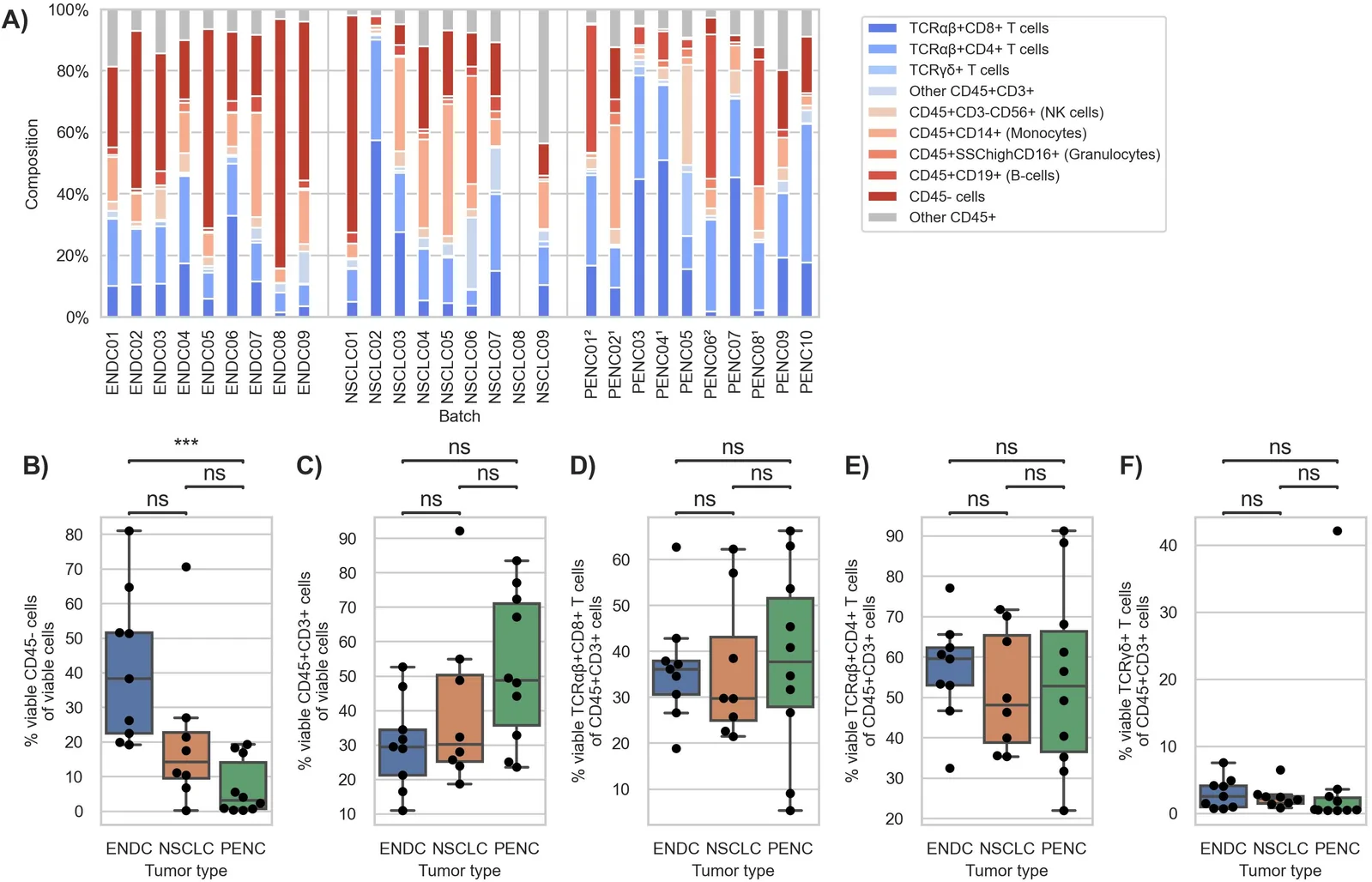
Tumor digest cell composition characterization using flow cytometry **(A)** Overview of cell compositions per tumor digest. 1 = PENC tumor starting material derived from a lymph node metastasis. 2 = PENC tumor starting material derived from a mix of a lymph node metastasis and the primary tumor. Comparisons per tumor type of **(B)** % CD45- (tumor) cells of viable cells. **(C)** % CD45+CD3+ cells of viable cells; Of CD45+CD3+ T cells: **(D)** % TCRαβ+CD8+ T cells, **(E)** % TCRαβ+CD4+ T cells, and **(F)** % TCRγδ+ T cells. Boxplots show the median and interquartile range (IQR); whiskers extend to 1.5 × IQR, and points beyond this range are shown as outliers. Individual observations per batch are overlaid as points. Statistical analysis was performed using ANOVA or the Kruskal-Wallis test, with Tukey’s honest significant difference (HSD) test and Dunn’s multiple comparisons test, respectively. When ANOVA or the Kruskal-Wallis tests were non-significant (ns; p≥0.05), ns was annotated for all multiple comparisons. When significant, multiple comparisons were annotated as follows: ns, p≥0.05; ***p<0.001. ENDC, Endometrial carcinoma; NSCLC, Non-small cell lung cancer; PENC, Penile cancer.

As CD45+CD3+ cells are the active substance of NTU101, the composition of CD45+CD3+ cells and of their subsets in the tumor digest was investigated in detail. The tumor digests contained viable CD45+CD3+ cells with comparable median percentages of viable cells across the tumor types (ENDC, 29.5%; NSCLC, 30.2%; PENC, 48.8%; p=0.08, **Figure 2C**), with an overall range from 11.0%–92.2% (**Supplementary Table 6**). These percentages were comparable to those observed in metastatic melanoma-derived tumor digests used to manufacture TILs (37). Within the CD45+CD3+ cell subset, the median percentage of TCRαβ+CD8+ T cells was 36.1% in ENDC, 29.7% in NSCLC, and 37.7% in PENC, with values ranging from 5.3%–66.2% (**Figure 2D**; **Supplementary Table 5**). TCRαβ+CD4+ T cells made up a slightly higher percentage of CD45+CD3+ cells, with a median of 59.5% in ENDC, 48.1% in NSCLC, and 52.8% in PENC, and a range of 21.9%–91.3% (**Figure 2E**; **Supplementary Table 5**). Of the CD4+ T cells, Tregs made up a median of 13.4%, ranging from 0.0%–44.6% (**Supplementary Figure 6**; **Supplementary Table 7**). Lastly, TCRγδ+ T cells generally represented a minor CD45+CD3+ cell subset, with median proportions of 2.5% in ENDC, 2.2% in NSCLC, and 0.6% in PENC, ranging from 0.4%–41.2% (**Figure 2F**; **Supplementary Table 5**). None of the differences in T-cell subset compositions were statistically significant between tumor types (**Figures 2D–F**).

The proliferation step was initiated using the tumor digest, and cultures were monitored throughout the manufacturing step. Using a brightfield microscope and hemocytometer, viable non-tumor cell proliferation was monitored. The non-tumor cells successfully proliferated from the tumor digests in all PD proliferation steps (**Figures 3A–C**). The cell composition of the resulting NTU101 intermediate products was evaluated by flow cytometry. NTU101 intermediate products consisted mostly of CD45+CD3+ cells, with the primary remaining non–T–cell population being CD45+CD3-CD56+ natural killer (NK) cells, and occasionally smaller cell populations of CD45- cells or immune cells observed in the tumor digest (**Figure 3D**; **Supplementary Table 5**). Overall, CD45+CD3+ cells expanded 6.2-fold–110.1-fold in the proliferation step with comparable medians across the tumor types (ENDC: 21.6, NSCLC: 37.8, PENC: 22.9; p=0.53; **Figure 3E**; **Supplementary Table 6**). This resulted in comparable median numbers of viable CD45+CD3+ cells between tumor types in the resulting NTU101 intermediate products (ENDC: 53.9×10^6, NSCLC: 51.4×10^6, PENC: 26.9×10^6; p=0.66; **Figure 3F**; **Supplementary Table 6**).

**Figure 3:**
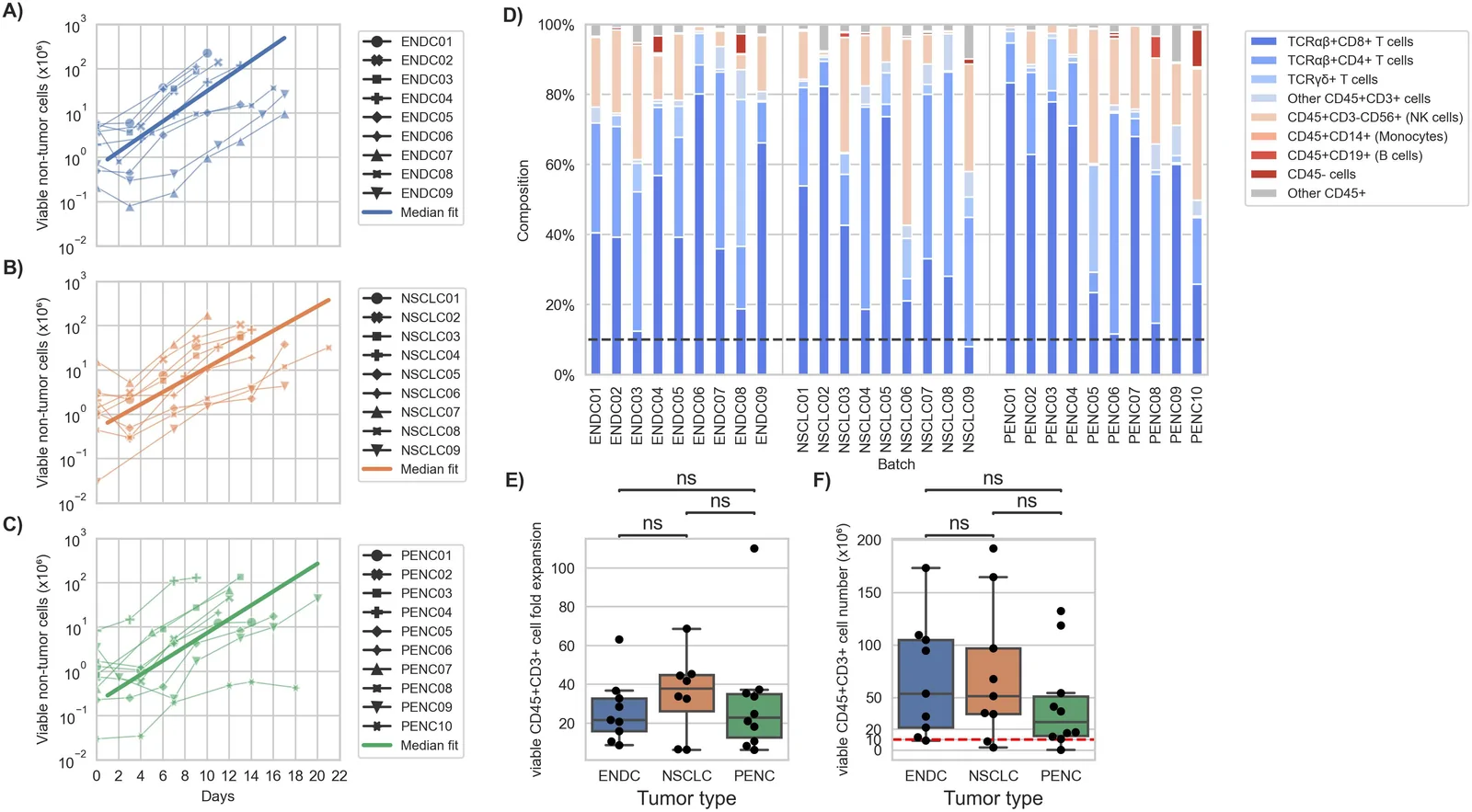
Proliferation step data for process development NTU101 runs. Viable non-tumor cells throughout the proliferation step of **(A)** Endometrial carcinoma (ENDC) runs, **(B)** Non-small cell lung cancer (NSCLC) runs, and **(C)** Penile cancer (PENC) runs. Thin lines show individual batches; the bold line indicates the median fitted growth trajectory. Growth rates were determined per run by linear regression of log_10_-transformed viable cell number on culture day, from day 1 onward. The bold line is defined by the median intercept across runs and the slope corresponding to the median doubling time. **(D)** Overview of cell compositions in the (end of proliferation) NTU101 intermediate product determined by flow cytometry (black dotted line: IPC2 ≥ 10% viable CD45+CD3+ cells). **(E)** Viable CD45+CD3+ cell fold expansion in the proliferation step. **(F)** Viable CD45+CD3+ cell number in the NTU101 intermediate product per tumor type (red dotted line: IPC3 ≥ 10×10^6 viable CD45+CD3+ cells). Boxplots show the median and interquartile range (IQR); whiskers extend to 1.5 × IQR, and points beyond this range are shown as outliers. Individual observations per run are overlaid as points. Statistical analysis was performed using ANOVA or the Kruskal-Wallis test, with Tukey’s honest significant difference (HSD) test and Dunn’s multiple comparisons test, respectively. When ANOVA or the Kruskal-Wallis tests were non-significant (ns; p≥0.05), ns was annotated for all multiple comparisons.

The impact of the tumor digest’s initial cell composition across the tumor types on expansion during the proliferation step and resulting CD45+CD3+ cell yield was investigated. When evaluating the tumor digest’s initial composition of T-cell subsets, higher non-tumor cell fold expansion was associated with higher initial percentages of TCRαβ+CD8+ (Spearman’s ρ=0.40 and p=0.041) and TCRγδ+ T cells (Spearman’s ρ=0.40 and p=0.038, **Supplementary Figure 7**). Other than these T-cell subsets, none of the tumor digest’s cell composition attributes were significantly associated with non-tumor cell fold expansion (**Supplementary Figure 7**). Higher initial percentages of TCRαβ+ CD8+ T cells (Spearman’s ρ=0.39 and p=0.044) and absolute CD45+CD3+ cell numbers (Spearman’s ρ=0.79 and p<0.001) were positively associated with a higher CD45+CD3+ cell yield (**Supplementary Figure 7**).

NTU101 intermediate products were within the IPC action limit ranges in all but four PD runs (n = 24/28, 86%; **Supplementary Table 6**). Intermediate products in these four runs failed to meet the 10×10^6 viable CD45+CD3+ cell number action limit (IPC3; ENDC07, NSCLC06, NSCLC09, and PENC10; **Supplementary Table 6**). Three out of four (excluding NSCLC06) had tumor starting material volumes that were <0.4 cm³ after trimming, and in two cases, the tissue was considered too small to quantify volumes accurately (ENDC07 and NSCLC09; **Supplementary Table 6**). Intermediate products that met IPC3 had trimmed median tumor starting material volumes of 4.5 cm³ and ranged from 0.13–36 cm³ (**Supplementary Table 6**). Small tumor volumes of the three small tumors that failed to meet IPC3 were associated with low viable CD45+CD3+ cell numbers at the start of proliferation (range: 0.02×10^6–0.1×10^6, **Supplementary Table 6**), which likely reduced the probability of achieving the 10×10^6 target number of CD45+CD3+ cells for the intermediate product. Five intermediate products that met IPC3 also started the proliferation with <1×10^6 viable CD45+CD3+ cells (NSCLC03, PEN01, PEN06, PEN08), with a minimum of ≥0.3×10^6 (END05, **Supplementary Table 6**). However, the probability of meeting IPC action limits is likely not zero when starting with <0.3×10^6 viable CD45+CD3+ cells, based on the observed variability in CD45+CD3+ cell fold expansion during the proliferation step (**Figure 3E**; **Supplementary Table 6**). Nonetheless, larger starting material volumes and viable cell numbers likely improve the manufacturing success rate. This is consistent with prior observations in melanoma-derived TIL manufacturing, where small-volume tumors retain the capacity to expand successfully in the proliferation step, albeit at a reduced probability of success (34, 38).

Cryopreserved NTU101 intermediate products were thawed and expanded in PD REPs. REPs were initiated using 0.1×10^6–1.7×10^6 viable CD45+CD3+ cells (**Supplementary Table 6**), with a target of around 1×10^6 cells. Cells were monitored using a brightfield microscope and hemocytometer, where viable cell growth was observed in all PD REPs (**Figures 4A–C**). At day 6 of the REP, viable cells had expanded >10-fold in all runs (IPC9, **Supplementary Table 6**). Over the full 14-day REP period, viable cells expanded 429 to 4925-fold (NSCLC06 and PENC10, respectively; **Supplementary Table 6**) and viable CD45+CD3+ cells expanded 668 to 9812-fold (ENDC07 and PENC10, respectively, **Supplementary Table 6**), with comparable medians across the tumor types (ENDC: 1114-fold, NSCLC: 1230-fold; PENC: 1469-fold; p=0.38; **Figure 4D**; **Supplementary Table 6**).

**Figure 4:**
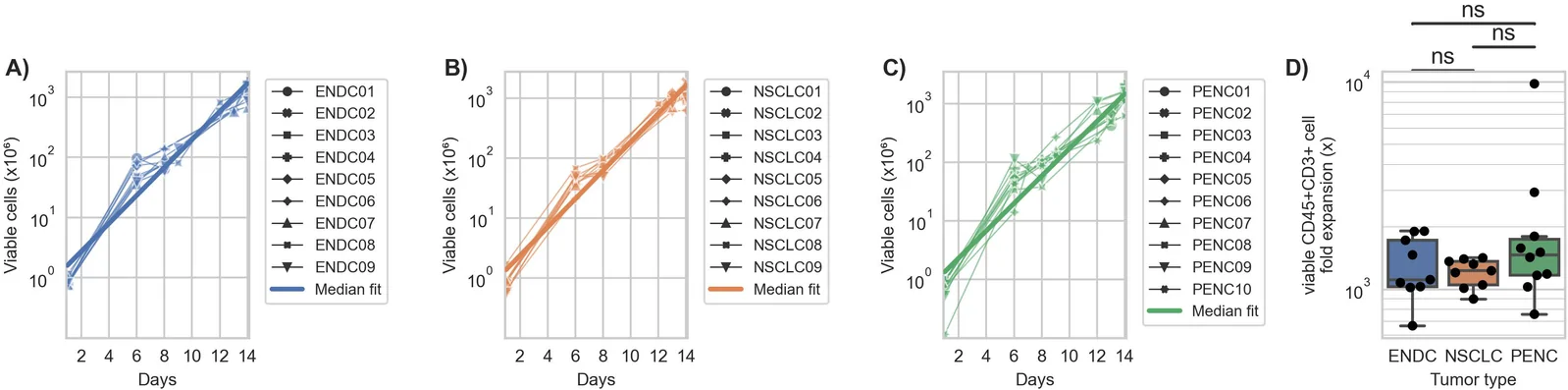
Rapid expansion phase (REP) data for process development NTU101 batches. Viable cell counts throughout the REP of **(A)** Endometrial carcinoma (ENDC) runs, **(B)** Non-small cell lung cancer (NSCLC) runs, and **(C)** Penile cancer (PENC) runs. Thin lines show individual batches; the bold line indicates the median fitted growth trajectory. Growth rates were determined per run by linear regression of log_10_-transformed viable cell number on culture day, from day 1 onward. The bold line is defined by the median intercept across runs and the slope corresponding to the median doubling time. **(D)** Viable CD45+CD3+ cell fold expansion in the REP, per tumor type and run. Horizontal grid lines indicate increments of 1,000-fold expansion. The boxplot shows the median and interquartile range (IQR); whiskers extend to 1.5 × IQR, and points beyond this range are shown as outliers. Individual observations per run are overlaid as points. Statistical analysis was performed using ANOVA or the Kruskal-Wallis test, with Tukey’s honest significant difference (HSD) test and Dunn’s multiple comparisons test, respectively. When ANOVA or the Kruskal-Wallis tests were non-significant (ns), ns was annotated for all multiple comparisons.

After harvest and formulation, PD NTU101-DP’s conformance with the RT acceptance criteria was evaluated. PD NTU101-DPs had 93.8–99.8% cell viability and 95.1–99.8% viable CD45+CD3+ cell purity, which met the RT acceptance criteria (**Figures 5A, B**; **Supplementary Table 8**). The percentage of CD45- cells of viable cells was consistently below the lower limit of quantification (LLOQ: <1%) and was undetectable (<0.2%) in 17 of 28 batches (**Supplementary Table 8**). CD19+ B cells were only detected (>0.2% of viable cells) in NSCLC 6 and PENC 9, and their percentage of viable cells was consistently below the LLOQ (<1%; **Supplementary Table 8**). Thus, the cellular impurity acceptance criteria were also consistently met (RT4: <1% and RT5: <1%, respectively). When compensated for the 1/20^th^ scale of the PD REP, all but one batch (PENC08) met the ≥10×10^9 viable CD45+CD3+ cell dose acceptance criterion (**Figure 5C**; **Supplementary Table 8**). 26 of 28 batches (excluding NSCLC04 and NSCLC08, **Supplementary Table 8**) secreted ≥1.0×10^3 pg/mL IFN-γ upon CD3/CD28-polymer stimulation (**Figure 5D**; **Supplementary Table 8**) and were therefore considered potent, demonstrating the presence of biologically active T cells capable of being triggered via their CD3/T-cell receptor (TCR) complex. None of the differences in RT values were statistically significant between TIL products derived from the three tumor types. Altogether, 89% (n=25/28) of PD NTU101-DPs met the evaluated RT acceptance criteria, with comparable RT attribute values across the tumor types (**Supplementary Table 8**; **Figure 5**).

**Figure 5:**
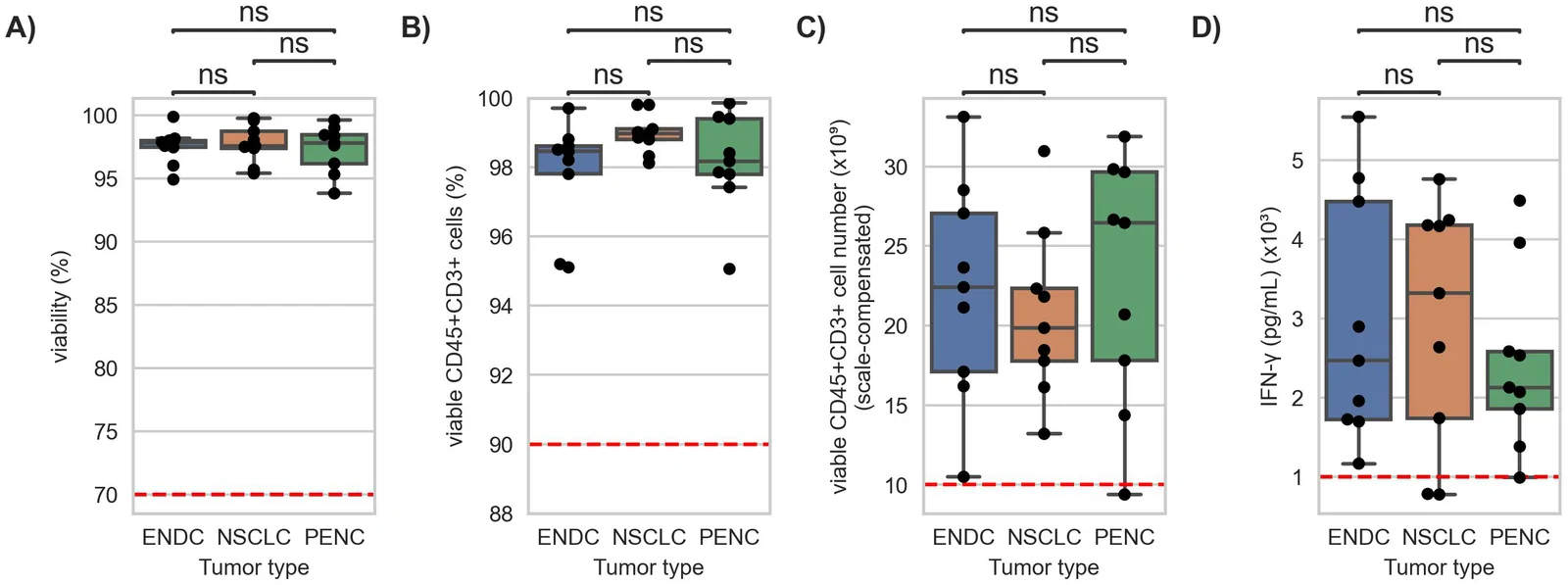
Release test (RT) data for process development (PD) NTU101 drug products (DPs) per tumor type. **(A)** Cell viability (red dotted line: RT8 ≥ 70% viable cells), **(B)** Viable CD45+CD3+ cell percentage determined by flow cytometry (red dotted line: RT3 ≥ 90% Viable CD45+CD3+ cells of viable cells), **(C)** viable CD45+CD3+ cell number (red dotted line: RT9 ≥ 10×10^9 viable CD45+CD3+ cells), compensated for the reduced scale of PD runs compared to GMP NTU101 manufacturing (multiplied 20-fold, to compensate for the 1/20^th^ scale), **(D)** Potency (IFN-γ release upon CD3/CD28-polymer stimulation) (red dotted line: RT1 ≥ 1×10^3 pg/mL IFN-γ). Boxplots show the median and interquartile range (IQR); whiskers extend to 1.5 × IQR, and points beyond this range are shown as outliers. Individual observations per batch are overlaid as points. Statistical analysis was performed using ANOVA or the Kruskal-Wallis test, with Tukey’s honest significant difference (HSD) test and Dunn’s multiple comparisons test, respectively. When ANOVA or the Kruskal-Wallis tests were non-significant (ns; p≥0.05), ns was annotated for all multiple comparisons. ENDC, Endometrial carcinoma; NSCLC, Non-small cell lung cancer; PENC, Penile cancer.

PD NTU101-DPs were characterized using flow cytometry to investigate whether TIL characteristics differed when derived from the different tumor types, by comparing the content of cellular impurities without RT acceptance criteria, T-cell subsets, and T-cell differentiation states. The cellular impurities observed in the NTU101 intermediate products (**Figure 3D**) were largely gone in all PD NTU101-DPs, although a few percent of NK cells remained in some cases (**Figure 6A**; **Supplementary Table 5**). The T-cell subsets in NTU101-DPs were highly variable, with PEN-derived PD NTU101-DPs trending towards having a higher percentage of TCRαβ+CD8+ T cells of viable cells compared to ENDC and NSCLC-derived PD NTU101-DPs, but the differences were not statistically significant (p=0.07; **Figure 6B**). During the proliferation step, the percentage of TCRαβ+CD8+ T cells of viable CD45+CD3+ cells increased (tumor digest vs NTU101 intermediate product, paired t-test p=0.0002), and this preferential expansion was often preserved in the NTU101-DP (tumor digest vs NTU101-DP, paired t-test p<0.0001; **Figure 6C**). Due to this, 89% (n=24/27) of NTU101-DPs were enriched for TCRαβ+CD8+ T cells compared to the tumor digest (**Supplementary Table 5**). Any difference in TCRαβ+CD4+ or TCRγδ+ T cells of total viable cells was not large enough to be statistically significant between PD NTU101-DPs derived from the three tumor types (**Figures 6D, E**). Analysis of T-cell differentiation status in NTU101-DP showed that in all but one NTU101 batch (NSCLC09), CD8+ and CD4+ T cells were mostly in a CD45RA-CCR7- (Effector memory) state, with a minority in a CD45RA-CCR7+ (Central Memory) state (**Figures 6F, G**; **Supplementary Table 9**). Some PD NTU101-DPs also contained a few percent of CD8+ and CD4+ T cells in a CD27-CD45RA+CD95+ (Effector) and/or CD27+CD45RA+ (naive-like) state (**Supplementary Table 9**). Altogether, the characteristics of the cellular impurities without RT acceptance criteria, CD45+CD3+ T-cell subsets, and T-cell differentiation states in PD NTU101-DPs were considered comparable when derived from the three tumor types.

**Figure 6:**
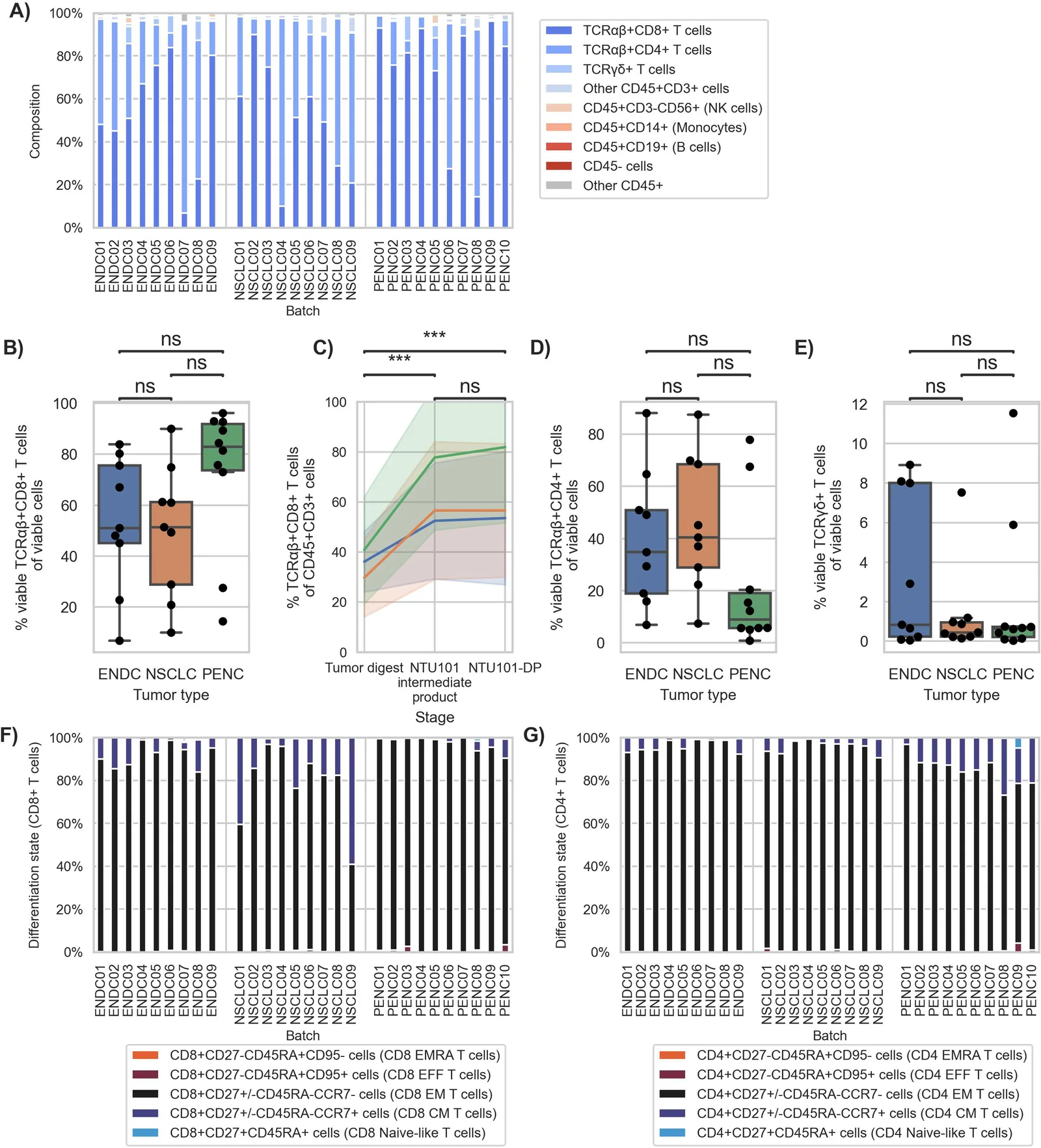
Cell composition and T-cell differentiation state data of process development NTU101-drug products (DPs) and change in TCRαβ+CD8+ T cells composition during manufacturing steps. **(A)** Cell composition overview determined by flow cytometry in NTU101-DPs. Percentage of viable TCRαβ+CD8+ T cells of viable cells **(B)** in NTU101-DPs. **(C)** Mean percentage viable TCRαβ+CD8+ T cells of CD45+CD3+ cells in: tumor digest, (end-of-proliferation) NTU101 intermediate products, and NTU101-DP, compared with a paired t-test, with confidence intervals representing the standard deviation at each stage. Blue = ENDC, Orange = NSCLC. Green = PENC. Paired t-test results were annotated as follows: non-significant (ns), p≥0.05; ***p<0.001. Percentage of **(D)** TCRαβ+CD4+ T cells of total viable cells, and **(E)** TCRγδ+ T cells of total viable cells. Boxplots show the median and interquartile range (IQR); whiskers extend to 1.5 × IQR, and points beyond this range are shown as outliers. Individual observations per batch are overlaid as points. Statistical analysis was performed using ANOVA or the Kruskal-Wallis test, with Tukey’s honest significant difference (HSD) test and Dunn’s multiple comparisons test, respectively. When ANOVA or the Kruskal-Wallis tests were ns (p≥0.05), ns was annotated for all multiple comparisons. When significant, multiple comparisons were annotated as follows: ns, p≥0.05; *p<0.05, **p<0.001, ***p<0.0001. T-cell differentiation state data of **(F)** CD8+ T cells and **(G)** CD4+ T cells in NTU101-DPs. ENDC, Endometrial carcinoma; NSCLC, Non-small cell lung cancer; PENC, Penile cancer; EMRA, effector memory CD45RA+; EFF, effector; EM, effector memory; CM, central memory.

The impact of PD NTU101-DP attributes on the IFN-γ release upon CD3/CD28-polymer stimulation was investigated. Across NTU101 T-cell subset and differentiation state attributes, higher percentages of CM CD8+ T cells (Spearman’s ρ= 0.43 and p=0.021) were positively associated with IFN-γ release (**Supplementary Figure 8**). This association could be explained by higher average expression of CD28 on CM CD8+ T cells compared to EM CD8+ T cells (39, 40), possibly making CM CD8+ T cells more responsive to the CD3/CD28-based stimulation. There was no significant association between NTU101 cell viability, number of viable CD45+CD3+ cells, or CD45+CD3+ fold expansion in the REP and IFN-γ release (**Supplementary Figure 8**).

To investigate the *in vitro* tumor-reactivity of PD NTU101 intermediate products and DPs to autologous tumor cells, cytotoxicity and/or IFN-γ secretion upon co-culture with autologous tumor digest cells were assessed according to the predefined criteria (see Materials and Methods). Cytotoxicity was observed in 69% of co-cultures with tested NTU101 intermediate products (ENDC: 88%, n=7/8; NSCLC: 75%, n=3/4; PEN: 25%, n=1/4; **Supplementary Figures 9A–C** where intermediate products considered tumor-reactive are outlined in black), while an increase IFN-γ secretion of at least 1.5-fold was detected in 67% of tested co-cultures (ENDC: 40%; n=2/5; NSCLC: 67%, n=2/3; PEN: 100%, n=4/4; **Supplementary Figures 9D–F** where intermediate products considered tumor-reactive are outlined in black). Overall, 94% of evaluated NTU101 intermediate products were considered tumor-reactive (END: 88%, n=7/8; NSCLC: 100%, n=4/4; PEN: 80%, n=4/5). Most importantly, cytotoxicity was observed in 60% of co-cultures with tested NTU101-DPs (ENDC: 75%; n=4/6; NSCLC: 60% n=3/5; PEN: 50%, n=2/4; **Figures 7A–C**), while specific IFN-γ production was detected in 92% of co-cultures (ENDC: 83%; n=5/6; NSCLC: 100% n=4/4; PEN: 100%, n=4/4; **Figures 7D–F**). Therefore, 82% of tested NTU101-DPs were considered tumor-reactive (END: 71%, n=5/7; NSCLC: 80%, n= 4/5; and PEN: 100%, n=5/5).

**Figure 7:**
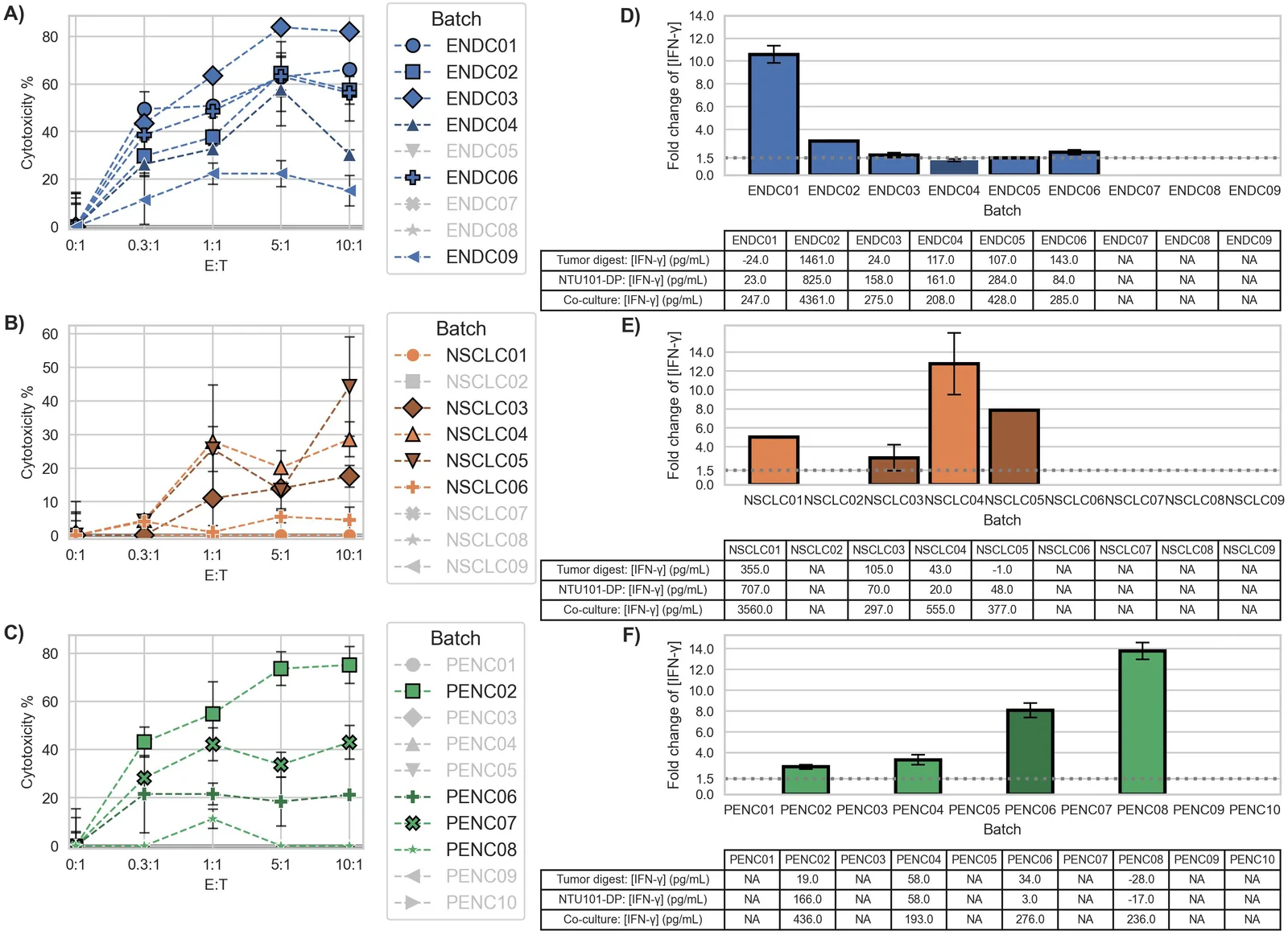
*In vitro* tumor-reactivity of process development NTU101 drug products (DPs). Cytotoxicity (relative cell killing) of CD45- target cells (T) within an autologous tumor digest, following co-culture with NTU101-DP effector cells **(E)** at increasing E:T ratios. Tumor digest alone (E:T = 0:1) serves as the negative control and internal standard. Cytotoxicity data for **(A)** Endometrial carcinoma (ENDC)-derived DPs, **(B)** Non-small cell lung cancer (NSCLC)-derived DPs, and **(C)** Penile cancer (PENC)-derived DPs. For greyed-out batches in the legend, there were insufficient viable cells available for co-culture or CD45- cells in the tumor digest. Fold increase of (absolute) IFN-γ concentration upon NTU101-DP cells (Responder; R) co-culture with tumor digest (Stimulator; S) in a 1:5 R:S ratio, compared to the highest concentration of NTU101-DP cells or tumor digest cells alone of **(D)** ENDC batches, **(E)** NSCLC batches, and **(F)** PENC batches. The gray dotted line represents the 1.5-fold increase in IFN-γ concentration required to identify tumor-reactive NTU101-DPs. The average of IFN-γ concentrations (pg/mL), measured in triplicate, is reported for each batch under the bar plots per condition. In both the line and bar plots, drug products considered tumor-reactive are outlined in black. In all panels, error bars represent the standard deviation of the replicates. For the NC condition, error bars represent the %CV of the NC replicates, which serves as the normalization reference for all other conditions. Darker markers and bars represent NTU101-DPs derived from mismatch repair-deficient and HPV-positive starting materials for ENDC and PENC, respectively, and NTU101-DPs derived from PD-L1-positive NSCLC starting materials. All tested NTU101-DPs were derived from NSCLC starting materials from smokers. In the enumeration of the tumor digest to determine E:T and R:S ratios, no distinction was made between viable tumor and non-tumor cells. Due to the presence of viable non-tumor cells in the tumor digest, the E:T and R:S cell ratios were likely higher than the reported ratios.

Interestingly, tumor-reactive NTU101-DPs were successfully derived from MMR-proficient ENDC tumors and HPV-negative PENC tumors (**Figures 7A, C, D** and **F**). Furthermore, whilst all tested NSCLC-derived NTU101-DPs were from smokers, both PD-L1-positive and negative tumors yielded tumor-reactive NTU101-DPs (**Figures 7B, E**). Overall, these results demonstrate that tumor-reactive NTU101-DPs can be generated from excised END, NSCLC, and PENC tumor starting materials with diverse biological characteristics. This builds upon previous results that demonstrated tumor-reactive NSCLC-derived TILs can be generated independently of metastasis locations for the starting material or patient treatment histories (25).

PD manufacturing experience demonstrated that NTU101-DPs could be manufactured that meet TM001’s quality criteria. Therefore, it was considered valid to implement of most of these quality criteria for determining the quality of GMP NTU101 batches (**Supplementary Table 1**). The exception was RT1, potency. One of the NTU101-DPs that failed to meet the predefined potency acceptance criterion (NSCLC04, **Supplementary Table 8**) was considered tumor-reactive (**Figures 7B, E**). This suggested that implementing TM001’s potency RT acceptance criterion in the NTU101 platform may result in the exclusion of potentially efficacious batches. Therefore, NTU101’s potency acceptance criterion was lowered to ≥0.5×10^3 pg/mL.

### 3.3 GMP NTU101 batch results

To confirm the NTU101 platform’s suitability for manufacturing TILs tumor-agnostically, one batch per tumor type was manufactured using the GMP manufacturing process (patient and material characteristics are presented in **Table 2**). The three NTU101-DPs met all quality criteria (IPC data described in **Supplementary Table 10** and RT data described in **Table 3**). In the GMP REPs, viable cells expanded 2902-, 2583-, and 1909-fold, respectively, for the END-, NSCLC-, and PEN-derived batches, whilst viable CD45+CD3+ cells expanded 3200-, 3100-, and 2100-fold, respectively (**Supplementary Table 10**). This was around 2- to 3-fold the median expansion of the PD REPs (**Figure 4D**) and resulted in viable CD45+CD3+ doses of 42–64×10^9 (RT9: ≥10×10^9), with 95–97% viability (RT8: ≥70%; **Table 3**). GMP NTU101-DPs contained 98.8–99.8% CD45+CD3+ cells (RT3: ≥90%) and less than 1% of cellular impurities (CD45- and CD19+ cells; RT4 and RT5, respectively; **Table 3**). GMP NTU101-DPs were free of microbial contamination (Sterility, RT2: No growth; Mycoplasma, RT6: not detected; and Endotoxins, RT10: ≤ 1.2 EU/mL) and met the appearance specification (RT7: Appearance; **Table 3**). Finally, NTU101-DPs were potent, secreting 0.8–5.9×10^3 pg/mL IFN-γ (RT1: ≥0.5×10^3; **Table 3**). Unfortunately, the tumor reactivity of GMP NTU101-DP could not be evaluated as no residual autologous tumor digest was available.

**Table 3:** Release test data of GMP NTU101 batches.

Release test (RT)	Acceptance criteria	NTU101-ENDC	NTU101-NSCLC	NTU101-PENC
RT1: Potency	≥0.5×10^3 pg/mL IFN-γ	3.7×10^3	0.8×10^3	5.9×10^3
RT2: Sterility	No Growth	no growth	no growth	no growth
RT3: Viable CD45+CD3+ cells (%)	≥90%	99.1	99.8	98.8
RT4: Viable CD45- cells (%)	<1%	<LOD	<LLOQ	<LOD
RT5: Viable CD19+ cells (%)	<1%	<LOD	<LOD	<LOD
RT6: Mycoplasma	Not detected	Not detected	Not detected	Not detected
RT7: Appearance	Drug product in the infusion bag. The bag is clean and intact, and there is no sign of leaks. Cell suspension white to off-white turbid liquid. No foreign particles or clumps.	Complies	Complies	Complies
RT8: Viability (%)	≥70%	97	96	95
RT9: Viable CD45+CD3+ cells	≥10×10^9	64.0×10^9	62.0×10^9	42.0×10^9
RT10: Endotoxins	≤1.2 EU/mL	<0.8	<0.8	<0.8

ENDC, Endometrial carcinoma;NSCLC, Non-small cell lung cancer;PENC, Penile cancer;LLOQ, Lower limit of quantification (<1%);LOD, Limit of detection (<0.2%).

## 4 Discussion

This study assessed whether the NTU101 platform, using one uniform manufacturing process, QC, and release strategy, could be used to manufacture TILs tumor-agnostically from excised ENDC, NSCLC, and PENC lesions. Of the 28 excised tumor lesions processed during PD, 25 (89%) NTU101-DPs met the predefined RT acceptance criteria, and 14 of 17 (82%) were considered tumor-reactive. PD NTU101-DPs derived from the different tumor types had comparable cell composition and T-cell differentiation state characteristics. Importantly, PD data justified lowering the potency acceptance criterion for GMP NTU101 manufacturing to ensure potentially efficacious batches are not rejected in batch release. All three GMP NTU101 batches met the revised quality criteria, confirming the suitability of the manufacturing process and control strategy. Together, these findings indicate that the NTU101 platform can robustly generate tumor-reactive TILs, with comparable quality attributes.

Based on their biological characteristics, most ENDC and PENC tumor starting materials were expected to have a low TMB (18, 19, 21). However, 71% and 100% of the derived PD NTU101-DPs, respectively, were considered tumor-reactive. In metastatic melanoma-derived TIL products, tumor-reactivity assays detected tumor-reactivity in 67–70% of tested TIL products (34, 41), where the tumor starting material can be expected to have a high TMB (21). Directly comparing these results with the NTU101 study should be done with care, due to technical differences between the assays and the small sample sizes. Nevertheless, these findings challenge the notion that a high TMB is an important tumor starting material characteristic for TIL manufacturing and clinical outcomes, as reported by some studies (42, 43), but contradicted by others (44–46). The NTU101 platform’s robustness in generating potentially tumor-reactive PD NTU101 DPs, independent of the tumor’s TMB, supports increasing the number of evaluated solid tumor types, including some that were previously thought to be unsuitable.

The NTU101 platform demonstrated a preferential outgrowth for TCRαβ+CD8+ T cells in 89% of PD NTU101 batches across tumor types. In addition, higher initial percentages of TCRαβ+CD8+ in the tumor digest were associated with higher expansion and CD45+CD3+ yields in the proliferation step. TIL products derived from ENDC, PENC, and NSCLC tumors in other studies contained CD8/CD4 ratios that were comparable to, or lower than (24, 26, 27, 29), what was observed in this study. This suggests that the NTU101 platform may preferentially expand TCRαβ+CD8+ T cells at levels comparable to, or higher than, other TIL manufacturing processes. The mechanism underlying the preferential expansion of TCRαβ+CD8+ T cells was not directly investigated but may relate to conditions in the proliferation step favoring CD8+ T-cell activation (47), potentially through HLA-I–mediated antigen presentation and differential responses to IL-2 compared to CD4+ T cells (48–51). The preferential expansion of TCRαβ+CD8+ T cells could aid NTU101’s clinical performance, as a higher number of TCRαβ+CD8+ T cells in metastatic melanoma-derived TIL products is positively associated with clinical efficacy (52–55). However, tumor-reactive CD4+ T cells in TIL products have also been reported (24, 56, 57). Therefore, the potential contribution of CD4+ T cells to clinical efficacy cannot be excluded. In addition, the positive association of high numbers of TCRαβ+CD8+ T cells with improved clinical performance when treating non-melanoma tumor types needs to be confirmed in the clinical setting.

In this study, a batch-release potency assay was chosen that allows rapid and reliable potency determination for all batches (58, 59), measuring IFN-γ release upon CD3/CD28-polymer stimulation. Such assays have been used as batch-release potency assays for other T-cell-based medicinal products, including TIL products (60, 61), as the readout is a surrogate measure of effector function of CD45+CD3+ cells after triggering the CD3/TCR complex (58, 62). By doing so, the potency assay meets the regulatory expectation of quantifying a relevant biological function related to the TIL product’s mechanism of action (MoA) (59) and can do so reliably for every NTU101 batch. As there is currently a lack of reliable alternatives (63), the chosen potency assay was considered fit-for-purpose for GMP NTU101 batch-release during potential upcoming clinical trials.

Due to the importance of tumor-reactive CD45+CD3+ cells for TIL product efficacy (22, 23), and tumor-reactivity likely being central to its MoA, some have proposed using co-culture assays with autologous tumor digest and TIL products as batch-release potency assays (64). However, in this study, these specific co-cultures were only feasible for 17 of 31 batches (54%), due to a lack of control of the number of viable cells and quality of the excised tumor, even though a potency readout will be required for batch-release of every single NTU101 batch (58, 59). Furthermore, whilst one study reported tumor-reactive TILs were positively associated with clinical responses (44) or that clinical responders all received TILs that were considered tumor-reactive (45), another found no correlation of tumor-reactivity with clinical outcomes (65). Thus, the association of tumor-reactive TILs with clinical efficacy has so far proven inconsistent. For these reasons, co-culture assays using autologous tumor digest were not deemed suitable for batch-release purposes of GMP NTU101-DPs, although co-culture assays enabled the identification of tumor-reactive cells in PD NTU101 batches, providing evidence that NTU101 products have efficacious potential.

Several limitations should be considered when interpreting these findings. The total number of NTU101 batches evaluated was limited, and tumor-reactive potential could not be evaluated for all products due to variability in the availability and quality of excised tumor material. Furthermore, the cytotoxicity assay employed a fixed number of CTV-stained tumor digest cells regardless of their CD45- fraction, resulting in an unknown and variable number of target cells per co-culture condition. Consequently, the percentage cytotoxicity values, particularly at lower CD45- percentages, may reflect changes in a small absolute number of events, limiting the statistical reliability of the assay and precluding quantitative comparisons of cytotoxic activity across batches. In addition, the mechanisms underlying the preferential expansion of tumor-reactive and TCRαβ+ CD8+ T cells during NTU101 manufacturing were not directly investigated. Unfortunately, we were also not able to quantify the number of Tregs in the final product, which were identified as CD127lowCD25+FoxP3+ in the tumor digest. Due to activation with IL-2, both FoxP3 and CD25 are naturally upregulated on conventional CD4 T cells as well, making it difficult to distinguish them from actual Tregs. In theory, Tregs could be present in the TIL product. However, the culture conditions used in TIL manufacturing are not beneficial for Tregs, as the presence of rapamycin, specifically engineered artificial feeder cells, and their initial isolation are generally required for effective Treg expansion (66). In addition, these conditions are also required for the stability of the Tregs’ immunosuppressive phenotype (67, 68). Due to the constitutive expression of CD25 by Tregs, low-dose (300 IU/mL) IL-2 is generally used; high-dose IL-2 (3000 IU/mL and 6000 IU/mL) is purposefully used in TIL manufacturing to preferentially expand non-Tregs (69, 70). Therefore, though not directly demonstrated in this study, these factors make it unlikely that meaningful expansion of Tregs has taken place that maintain an immunosuppressive phenotype into the TIL product. Future studies incorporating larger datasets and more detailed biological characterization during manufacturing will be important to validate these observations, but were considered outside of the scope of this study.

The NTU101 platform consistently generated TILs with comparable characteristics across tumor types, supporting its use in clinical trials. Here, a platform-based approach offers advantages over tumor-specific manufacturing, including faster trial initiation, reduced regulatory burden through reuse of core documentation, and lower development costs. Being a fresh cell therapy product, NTU101 will likely need to be manufactured using a decentralized manufacturing model to enable scalability. Here, standardization of the manufacturing process enables efficient technology transfer to new manufacturing sites and may facilitate extension of the NTU101 platform to additional tumor types, pending preclinical validation.

Implementing several process changes could improve future platform iterations, namely transferring the remaining open and manual steps to closed and, where possible, automated systems. In NTU101, the dynamic phase of the REP is already performed in a closed and automated bioreactor. Full closure of the REP is likely feasible. Automating the more operationally complex proliferation step could prove more challenging. An alternative would be to enrich for the tumor-reactive T cells directly from the tumor digest based on CD137 expression (71), or a combination of alternative markers (72). Such an approach could enrich the tumor-reactive T cell fraction in the final TIL product while potentially shortening the manufacturing time by eliminating the proliferation step (33). However, by shortening the manufacturing time, this approach may also yield fewer cells for infusion (33), and the clinical significance of administering fewer T cells, despite their enrichment for tumor-reactivity, remains unclear. Moreover, this enrichment strategy has primarily been evaluated in melanoma, and its applicability across other tumor types remains to be established (33). Given the preferential expansion of TCRαβ+CD8+ T cells observed during the NTU101 platform’s proliferation step across tumor types, the impact of eliminating this step on the critical characteristics of the final TIL product warrants further investigation.

There is currently limited consensus on the critical product characteristics for TIL products across tumor types, which determine their efficacy. First-in-human trials in non-melanoma indications will be essential to identify these characteristics, potentially in a tumor-specific manner. Linking NTU101 product characteristics to clinical outcomes will enable rational optimization, including evaluation of how manufacturing parameters influence these product characteristics. To do this effectively, more extensive characterization of starting material, intermediates, and final products will likely be required than was performed in this study. These steps align with the Quality-by-Design framework for pharmaceutical development and may support the development of more efficacious and consistent TIL products (73, 74). Importantly, however, many TIL product attributes are likely associated with tumor starting material attributes, which are inherently difficult (or even impossible) to control. Therefore, one should be cautious with defining strict acceptance criteria for starting material attributes. For example, prior to tumor resection, lesion size can be estimated by CT imaging, and lesions with at least a 2cm diameter are generally considered more likely to yield successful TIL outgrowth (34, 38). In contrast, other potentially relevant attributes, such as the degree of CD3+ T-cell infiltration, cannot be reliably assessed before surgical excision. Although these parameters can be quantified following tumor digestion, the patient has by then already undergone an invasive procedure. Given that successful TIL manufacturing remains possible even from tumors with low T-cell infiltration, it would be unethical to discontinue manufacturing solely on the basis of unfavorable post-resection characteristics.

In conclusion, this study demonstrated that the NTU101 platform can successfully manufacture tumor-reactive TILs from excised ENDC, NSCLC, and PENC lesions with comparable product characteristics across tumor types. Platform-based manufacturing reduces development timelines and costs and improves the scalability of TIL manufacturing by enabling decentralized manufacturing. Future studies will focus on clinical validation and further optimization of the platform, including the implementation of Quality-by-Design principles. If clinical efficacy is confirmed, the NTU101 platform may be extended to additional tumor types.

## Data Availability

The original contributions presented in the study are included in the article/**Supplementary Material**. Further inquiries can be directed to the corresponding authors.
