# Supplementary material for: Platform-based manufacturing of tumor-infiltrating lymphocytes derived from different solid tumor types
Source: Front Immunol. 2026 Oct 5;17:1854761. doi: 10.3389/fimmu.2026.1854761 (PMC13677931; doi:10.3389/fimmu.2026.1854761)
Supplement: Supplementary file 1 [file DataSheet1.docx]

Supplementary Figure 1 Gating strategy used to determine cell populations in the cell composition flow cytometry assay for tumor digest samples, applied to a representative sample (ENDC04).

Supplementary Figure 2 Gating strategy used to determine cell populations in the cell composition flow cytometry assay for NTU101 intermediate product and drug product samples, applied to a peripheral blood mononuclear cell (PBMC) assay control sample.

Supplementary Figure 3 Gating strategy used to determine cell populations in the T-reg flow cytometry assay applied to a representative tumor digest sample.

Supplementary Figure 4 Gating strategy used to determine cell populations in the T-cell differentiation state flow cytometry assay, applied to a peripheral blood mononuclear cell sample

Supplementary Figure 5 Gating strategy used to determine cytotoxicity against CD45- cells (including tumor cells) in the autologous tumor digest (Target; T) compared to tumor digest alone (NC), when co-cultured with NTU101-DP (Effector; E) in increasing ratios.

Supplementary Figure 6 Proportion of T-regs of CD4+ T cells in tumor digest. ENDC = Endometrial carcinoma, NSCLC = Non-small cell lung cancer, PENC = Penile cancer

Supplementary Figure 7 Heatmap showing Spearman's rank correlation coefficients (ρ) of tumor digest attributes (cellular composition percentages of total viable cells) versus proliferation step fold expansion and final cell CD45+CD3+ yield. Colors indicate the strength and direction of the correlation. Asterisks denote statistically significant correlations (*p < 0.05, **p < 0.01, ***p < 0.001).

Supplementary Figure 8 Heatmap showing Spearman's rank correlation coefficients (ρ) of process development NTU101-drug product (DP) attributes versus IFN-γ release upon CD3/CD28-polymer stimulation. Colors indicate the strength and direction of the correlation. Asterisks denote statistically significant correlations (*p < 0.05, **p < 0.01, ***p < 0.001).

Supplementary Figure 9 *In vitro* tumor-reactivity of process development NTU101 intermediate products. Cytotoxicity (relative cell killing) of CD45- target cells (T) within an autologous tumor digest, following co-culture with NTU101 intermediate product effector cells (E) at increasing E:T ratios. Tumor digest alone (E:T = 0:1) serves as the negative control and internal standard. Cytotoxicity data for A) Endometrial carcinoma (ENDC)-derived intermediate products, B) Non-small cell lung cancer (NSCLC)-derived intermediate products, and C) Penile cancer (PENC)-derived intermediate products. For greyed-out batches in the legend, there were insufficient viable cells available for co-culture or CD45- cells in the tumor digest. Fold increase of (absolute) IFN-γ concentration upon NTU101 intermediate product cell (Responder; R) co-culture with tumor digest cells (Stimulator; S) in a 1:5 R:S ratio, of D) ENDC batches, E) NSCLC batches, and F) PENC batches. The average of IFN-γ concentrations (pg/mL) measured in triplicate are reported for each batch under the barplots per condition. In both the line and bar plots, intermediate products considered tumor-reactive are outlined in black. In all panels, error bars represent the standard deviation of the replicates. For the NC condition, error bars represent the %CV of the NC replicates, which serves as the normalization reference for all other conditions. Darker markers and bars represent intermediate products derived from mismatch repair-deficient and HPV-positive starting materials for ENDC and PENC, respectively, and intermediate products derived from PD-L1-positive NSCLC starting materials. All tested intermediate products were derived from NSCLC starting materials from smokers. In the enumeration of the tumor digest to determine E:T and R:S ratios, no distinction was made between viable tumor and non-tumor cells. Due to the presence of viable non-tumor cells in the tumor digest, the E:T and R:S cell ratios were likely higher than the reported ratios
