# Supplementary material for: Platform-based manufacturing of tumor-infiltrating lymphocytes derived from different solid tumor types
Source: Front Immunol. 2026 Oct 5;17:1854761. doi: 10.3389/fimmu.2026.1854761 (PMC13677931; doi:10.3389/fimmu.2026.1854761)
Supplement: Supplementary file 2 [file Table1.docx]

Supplementary Table 1 Justifications for NTU101 quality criteria: release test (RT) acceptance criteria and in-process control (IPC) action limits. Quality criteria and justifications were initially derived from manufacturing experience with TM001, a TIL-MP used to treat patients with metastatic melanoma, and adapted for NTU101 based on process development batch data.

| Manufacturing step | Sampling time | Parameter | Analytical method | Quality criteria: GMP NTU101 (PD NTU101) | Justification |
| --- | --- | --- | --- | --- | --- |
| In-process controls (IPCs) | | | | | |
| Step 3a: Proliferation | End Day -1 | **IPC1:** Sterility | Ph. Eur. 2.6.27 (growth-based method) | No growth (NA) | NTU101 is manufactured under aseptic conditions, in line with ATMP GMP Guidelines (C(2017) 7694). To confirm that the received tumor starting material is sterile and that step 3a is performed under aseptic conditions, sterility is assessed. |
|  |  | **IPC2:** CD45+CD3+ cells (%) | Flow cytometry (cell composition panel) | ≥10.0% | For NTU101-DP to meet RT3 (≥ 90.0% CD45+CD3+ cells), the active substance must be successfully isolated from the received tumor starting material. To confirm the active substance was successfully isolated from the tumor starting material, the viable CD45+CD3+ cell purity is assessed. In PD NTU101 manufacturing, ≥42.2% viable CD45+CD3+ cell purity was measured at this timepoint, with significant variation observed between batches (Supplementary Table 6). An action limit of ≥10.0% viable CD45+CD3+ cells was set as a minimum threshold, which would allow expansion in the subsequent step 4, in line with TM001. |
|  | End | **IPC3:** Viable CD45+CD3+ cell number | Calculation | ≥10×10^6 cells | To ensure that RT9 (≥ 10×10^9 viable CD45+CD3+ cells) is consistently met, an IPC is applied. In PD NTU101 batches, CD45+CD3+ cells expanded > 1000-fold in 89% (25/28) (Supplementary Table 6). As a 1000-fold expansion in the subsequent REP is expected to be readily achievable, the action limit of ≥ 10×10^6 viable CD45+CD3+ cells was set, in line with TM001. |
|  |  | **IPC4:** viable non-tumor cell fold expansion | Calculation | >2-fold | Successful proliferation of viable CD45+CD3+ cells is expected in step 3a and indicates that the isolated viable CD45+CD3+ cells from the tumor starting material possess proliferative potential, a prerequisite for achieving RT9. Therefore, an action limit of >2-fold non-tumor cell expansion was set, in line with TM001. |
|  |  | **IPC5:** % viable tumor cells | Calculation | <10% | Lymphocytes isolated from the tumor starting material should be capable of eliminating tumor cells; successful tumor cell elimination at this stage indicates the isolated cell population possesses functional cytotoxic capacity and aids in ensuring RT4 (<1.0% viable CD45- cells, which include tumor cells) is consistently met. Therefore, the action limit was set at <10% viable tumor cells, in line with TM001. |
| Step 3b: Cryopreservation | Start | **IPC6:** Viable CD45+CD3+ cell number | Calculation | ≥80×10^6 cells (NA) | Cells cryopreserved in step 3b can undergo post-thaw apoptosis during the 24-hour recovery period prior to the initiation of step 4. To ensure that the target cell number required to start step 4 (IPC7) is consistently met following recovery, an action limit for cryopreservation of NTU101-intermediate product was set at ≥80×10^6 viable CD45+CD3+ cells prior to cryopreservation, in line with TM001. |
| Step 4: REP – Static (Day 1 - 6) | Start | **IPC7:** Viable CD45+CD3+ cell number | Calculation | ≥10×10^6 cells (≥0.5×10^6 cells^2^) | The same justification as IPC 3 applies to this action limit. When available, a target number of 20×10^6 viable CD45+CD3+ cells from the NTU101-intermediate product will be used to initiate step 4, in line with TM001. |
|  |  | **IPC8:** Ratio of viable feeders:viable CD45+CD3+ cells | Calculation | 200:1 ± 10% (100-200:1) | Irradiated feeder cells provide co-stimulation when activating viable CD45+CD3+ cells at the initiation of the REP, supporting the proliferative capacity required to reach RT 9. A viable feeder-to-viable CD45+CD3+ cell ratio of 200:1 ± 10% was implemented for NTU101 to ensure consistent CD45+CD3+ cell activation across batches. This ratio was standardized from the broader 100–200:1 range used during PD NTU101 manufacturing, narrowing the range to reduce batch-to-batch variability in activation. |
|  | Day 6 | **IPC9:** Viable cell fold expansion | Calculation | >10-fold | To assess successful activation of viable CD45+CD3+ cells at the initiation of the REP and ensure the dose RT acceptance criterion will be met (RT9), fold expansion of viable cells was calculated on REP day 6. All PD NTU101 batches expanded >10-fold by day 6 of the REP (Supplementary Table 6), consistent with the expectation that successful activation would result in >10-fold expansion by this time point for TM001. The action limit was therefore set at >10-fold. |
|  |  | **IPC10:** Feeder cell - proliferation | Calculation | <1.0-fold | On REP day 6, the absence of growth in a feeder cell control culture is assessed to confirm successful irradiation of the feeder cells and to minimize the likelihood of surviving allogeneic feeder cells in NTU101-DP, which could pose a safety risk if administered to the patient. An action limit of <1.0-fold expansion of feeder cells in the control flask was set to ensure irradiated feeder cells are not proliferating. |
|  |  | **IPC11:** Viable cell number | Ph. Eur. 2.7.29 | ≥0.75×10^9 cells (NA) | NTU101-DS is transferred to the Xuri W25 Bioreactor on day 6 of the REP, after which the cell culture volume is increased to 4700 mL. However, when the viable cell number in NTU101-DS is too low on day 6 of the REP, increasing the bioreactor volume to 4700 mL could dilute cells to concentrations that would negatively impact CD45+CD3+ cell expansion. Therefore, an action limit of ≥0.75×10^9 viable cells was set for filling the bioreactor to 4700 mL for TM001. When this limit is not met, the cell culture volume in the bioreactor is instead increased to 4700 L on day 7 or day 8, depending on the viable cell number, and was also applied to NTU101. |
| Release Tests (RTs) | | | | | |
| Step 5: REP – Dynamic (Day 6 - 14) | Start (Day 6)^1^ | **RT1:** Potency | ELISA | ≥0.5×10^3 pg/mL IFN-γ (≥ 1.0×10^3 pg/mL IFN-γ) | Potency testing is required and should be linked to the relevant biological properties of the product (ICH Q6B). Interferon-gamma (IFN-γ) release after T-cell receptor (TCR)-based stimulation is a proxy for the cytotoxic capacity of T cells and is a requirement for their anti-tumor functionality; IFN-γ release after TCR-based stimulation was therefore implemented as the potency assay. TM001's potency acceptance criterion was ≥1.0×10^3 pg/mL IFN-γ. However, one PD NTU101-DP (NSCLC04) was considered tumor reactive (Figure 7B) but secreted only 0.8×10^3 pg/mL IFN-γ (Supplementary Table 8). Applying TM001's potency acceptance criterion unmodified would risk excluding potentially efficacious NTU101 batches. Therefore, NTU101's potency criterion was lowered to ≥0.5×10³ pg/mL IFN-γ. |
|  |  | **RT2:** Sterility | Ph.Eur. 2.6.27 (growth-based method) | No growth (NA) | Sterility testing is a compendial requirement for cellular products (Ph. Eur. 2.6.27). TM001's sterility acceptance criterion was "no growth," consistent with pharmacopeial requirements, and was also applied to NTU101. To mitigate the risk of transferring contaminated material to the closed-system bioreactor, RT2 is sampled after the cell culture is transferred to the closed-system bioreactor. |
|  | End (Day 14) | **RT3:** Viable CD45+CD3+ cells (%) | Flow cytometry (cell composition panel) | ≥90.0% | Purity testing is required to characterize the identity and consistency of the active substance and to control process-related impurities (ICH Q6B). CD45+CD3+ cells are the active substance of NTU101. TM001's acceptance criterion required ≥90.0% of viable cells in the drug product to be CD45+CD3+ cells. All PD NTU101 batches met this acceptance criterion (supplementary Table 8); therefore, NTU101’s acceptance criterion is also set at ≥ 90.0 %. |
|  |  | **RT4:** Viable CD45- cells (%) | Flow cytometry (cell composition panel) | <1.0% | Purity/impurity testing is required to control for cell populations other than the intended active substance that may pose a risk to patient safety (ICH Q6B). The presence of viable CD45- cells, including residual tumor cells, could pose a potential safety risk for the patient; a maximum amount of viable CD45- cells in NTU101 is therefore defined to ensure no outgrowth of residual tumor cells. The acceptance criterion was set at the LLOQ of the flow cytometry assay <1.0%. |
|  |  | **RT5:** Viable CD19+ cells (%) | Flow cytometry (cell composition panel) | <1.0% | Purity/impurity testing is required to control for cell populations other than the intended active substance that may pose a risk to patient safety (ICH Q6B). The presence of expanding B-cells (e.g., upon transformation with Epstein-Barr virus; EBV) in NTU101 could pose a safety risk in the form of EBV-related B-cell malignancies; a maximum amount of viable CD19+ cells in NTU101 is therefore defined to ensure that no outgrowth of B-cells occurs during NTU101 manufacturing. The acceptance criterion was set at the LLOQ of the flow cytometry assay <1.0%. |
| Step 6: Harvest | Supernatant fraction: End | **RT6:** Mycoplasma | ddPCR | Not detected (NA) | Mycoplasma testing is a compendial requirement for parenteral products (Ph. Eur. 2.6.7). TM001's mycoplasma acceptance criterion was "not detected," consistent with pharmacopeial requirements, and also applies to NTU101. Mycoplasma data are generated for batch release retrospectively, post-infusion. |
| NTU101 Formulation and fill | NTU101 | **RT7:** Appearance | Visual inspection | Drug product in the infusion bag. The bag is clean and intact, and there is no sign of leaks. Cell suspension white to off-white turbid liquid. No foreign particles or clumps. (NA) | A qualitative statement describing the physical state, color, and clarity of the drug product is expected during batch release (ICH Q6B). The NTU101 cell dispersion for infusion is visually inspected for color and for the presence of particulate matter, either from foreign material, cellular (cell clumps/aggregates), or other precipitates. The bag is visually checked for signs of compromised closure integrity and the presence of irregularities. |
|  |  | **RT8:** Viability (%) | Ph. Eur. 2.7.29 | ≥70% | Cell viability testing is required to ensure the medicinal product consists predominantly of functional, living cells at release, since non-viable cells could compromise the product's efficacy or safety. The required percentage of viable cells in NTU101 is set in line with general industry practice for cellular therapies (≥70%). |
|  |  | **RT9:** Viable CD45+CD3+ cell number | Calculation | ≥10×10^9 cells (≥0.5×10^9 cells^2^) | The quantity or dose of the active substance in the drug product should be determined (ICH Q6B). A minimum viable CD45+CD3+ cell dose is expected to be required for NTU101 to be efficacious. TM001's viable CD45+CD3+ cell dose acceptance criterion was ≥ 10×10^9 cells. All but one PD NTU101 batch met this criterion when compensated for the 1/20^th^-scale of PD manufacturing (Supplementary Table 8). Thus, this dose acceptance criterion is expected to be consistently achievable and was also implemented for NTU101. |
|  |  | **RT10:** Endotoxins | Ph. Eur. 2.6.14 (chromogenic kinetic method) | ≤1.2 EU/mL^3^ (NA) | Bacterial endotoxins testing (chromogenic kinetic method) is a compendial requirement for parenteral products (Ph. Eur. 2.6.14). Bacterial endotoxin data are available for batch release post-infusion. To safeguard patient safety, the acceptance criteria for bacterial endotoxins are ≤1.2EU/mL^3^. |
|  |  | **RT11:** Sterility | Ph. Eur. 2.6.27 (growth-based method) | No growth (NA) | Sterility testing is a compendial requirement for parenteral products (Ph. Eur. 2.6.27). A sterility sample is taken from the NTU101 drug product, with the acceptance criterion set at "no growth," consistent with TM001. Given the turnaround time of the compendial sterility assay, data are generated for batch release retrospectively, post-infusion. |

GMP = good manufacturing practice, PD = Process development, ATMPs = Advanced therapeutic medicinal products, REP = rapid expansion phase, LLOQ = lower limit of quantification, ddPCR = digital droplet PCR, DP = drug product, 1 = To have a potency test result available prior to administration, the release tests are sampled after transferring the NTU101-DS to the closed-system bioreactor on day 6 of the REP. 2 = quality criteria were 1/20^th^ of the GMP NTU101 quality criteria, due to the 1/20^th^-scale of the PD REP. 3 = The bacterial endotoxins limit (EU/mL) for NTU101 was calculated by dividing the threshold of pyrogenic dose of endotoxin per kg of body mass (K) (5 EU/kg, as specified in the Ph.Eur. 5.1.10) by the maximum recommended dose of product per kg of body mass (M) (200mL). In this calculation, a patient body weight of 50kg is assumed, which is considered a worst-case scenario for an adult patient’s body weight.
