# Supplementary material for: Platform-based manufacturing of tumor-infiltrating lymphocytes derived from different solid tumor types
Source: Front Immunol. 2026 Oct 5;17:1854761. doi: 10.3389/fimmu.2026.1854761 (PMC13677931; doi:10.3389/fimmu.2026.1854761)
Supplement: Supplementary file 3 [file Table10.docx]

Supplementary Table 10 In-process control (IPC) and manufacturing data for GMP NTU101 runs

| Sampling time | In-process control (IPC) | Action limit | NTU101-ENDC | NTU101-NSCLC | NTU101-PENC |
| --- | --- | --- | --- | --- | --- |
| Step 3: Proliferation  (End Day -1) | **IPC1**: Sterility | No growth | no growth | no growth | no growth |
|  | **IPC2:** CD45+CD3+ cells (%) | ≥ 10.0 % | 90.40% | 84.30% | 87.40% |
| Step 3: Proliferation (End) | **IPC3:** Viable CD45+CD3+ cell number | ≥ 10×10^6^ cells | 309×10^6^ | 257×10^6^ | 192×10^6^ |
|  | **IPC4:** viable non-tumor cell fold expansion | > 2-fold | 22 | 22 | 169 |
|  | **IPC5:** % viable tumor cells | < 10 % | 0 | 0 | 0 |
| Step 3a: Cryopreservation | **IPC6:** Viable CD45+CD3+ cell number | ≥ 80×10^6^ cells | 457×10^6^ | 273×10^6^ | 134×10^6^ |
| Step 4a: Rapid expansion – Static phase (Day 1 – 6) (Start) | I**PC7:** Viable CD45+CD3+ cell number | ≥ 10×10^6^ cells | 20 | 20 | 20 |
|  | **IPC8:** Ratio of viable feeders:viable CD45+CD3+ cells | 200:1 ± 10% | 200 | 200 | 200 |
| Step 4a: Rapid expansion – Static phase (Day 1 – 6)  (Day 6) | **IPC9:** Viable cell fold expansion | > 10-fold | 84 | 79 | 68 |
|  | **IPC10:** Feeder cell - proliferation | < 1.0-fold | 0.5 | 0.55 | 0.75 |
|  | **IPC11:** Viable cell number | ≥ 0.75×10^9^ cells | 1.7×10^9^ | 1.6×10^9^ | 1.4×10^9^ |
| Step 4b: Rapid expansion – Dynamic phase (Day 6 – 14)  (Day 14) | Viable cell fold expansion | NA | 2909 | 2583 | 1909 |
|  | Viable CD45+CD3+ cell fold expansion | NA | 3200 | 3100 | 2100 |

NSCLC: Non-small cell lung cancer, PEN: Penile cancer, END: Endometrial carcinoma, NA= Not Applicable
