# Supplementary material for: Platform-based manufacturing of tumor-infiltrating lymphocytes derived from different solid tumor types
Source: Front Immunol. 2026 Oct 5;17:1854761. doi: 10.3389/fimmu.2026.1854761 (PMC13677931; doi:10.3389/fimmu.2026.1854761)
Supplement: Supplementary file 4 [file Table2.docx]

Supplementary Table 2 Cell composition panel antibodies

| Antibody | Supplier | Catalog Number |
| --- | --- | --- |
| PerCP-Cy5.5 anti-human CD3 antibody | BioLegend | 344807/344808 |
| APC anti-human CD45 antibody | BD Bioscience | 560973/555485 |
| BV711 anti-human CD19 antibody | BD Bioscience | 563038/563036 |
| BV510 anti-human CD4 antibody | BD Bioscience | 562971/562970 |
| AF700 anti-human CD8 antibody | BioLegend | 344723/344724 |
| BV605 anti-human CD56 antibody | BioLegend | 318333/318334 |
| BV421 anti-human TCRαβ antibody | BioLegend | 306721/306722 |
| PE anti-human TCRγδ antibody | BD Bioscience | 333141 |
| FITC anti-human CD14 antibody | BioLegend | 301803/301804 |
| PE-Cy7 anti-human CD16 antibody | BioLegend | 302015/302016 |
