# Supplementary material for: Platform-based manufacturing of tumor-infiltrating lymphocytes derived from different solid tumor types
Source: Front Immunol. 2026 Oct 5;17:1854761. doi: 10.3389/fimmu.2026.1854761 (PMC13677931; doi:10.3389/fimmu.2026.1854761)
Supplement: Supplementary file 5 [file Table3.docx]

Supplementary Table 3 T-reg composition panel antibodies

| Antibody | Supplier | Catalog Number |
| --- | --- | --- |
| PerCP-Cy5.5 anti-human CD3 antibody | BioLegend | 344807/344808 |
| APC anti-human CD45 antibody | BD Bioscience | 560973/555485 |
| BV510 anti-human CD4 antibody | BD Bioscience | 562971/562970 |
| AF700 anti-human CD8 antibody | BioLegend | 344723/344724 |
| PE-CF594 anti-human CD127 antibody | BD Bioscience | 562397 |
| PE anti-human CD25 antibody | BioLegend | 302606 |
| PE-Cy7 anti-human PD-1 antibody | BD Bioscience | 561272 |
| BV421 anti-human Foxp3 antibody | BioLegend | 320124 |
