# Supplementary material for: Platform-based manufacturing of tumor-infiltrating lymphocytes derived from different solid tumor types
Source: Front Immunol. 2026 Oct 5;17:1854761. doi: 10.3389/fimmu.2026.1854761 (PMC13677931; doi:10.3389/fimmu.2026.1854761)
Supplement: Supplementary file 6 [file Table4.docx]

Supplementary Table 4 T-cell differentiation state panel antibodies

| Antibody | Supplier | Catalog Number |
| --- | --- | --- |
| PerCP-Cy5.5 anti-human CD3 antibody | BioLegend | 344807/344808 |
| APC anti-human CD45 antibody | BD Bioscience | 560973/555485 |
| BV510 anti-human CD4 antibody | BD Bioscience | 562971/562970 |
| AF700 anti-human CD8 antibody | BioLegend | 344723/344724 |
| BV711 anti-human CD95 antibody | BioLegend | 305644 |
| BV650 anti-human CD27 antibody | BioLegend | 302827 |
| BV510 anti-human CD62L antibody | BioLegend | 304844 |
| FITC anti-human CD39 antibody | BD Bioscience | 561444 |
| PE-Cy7 anti-human CD28 antibody | BioLegend | 302925 |
| BV421 anti-human CCR7 antibody | BioLegend | 353207 |
| PE anti-human CD45RO antibody | BD Bioscience | 347967 |
| BV605 anti-human CD45RA antibody | BioLegend | 304134 |
| AF647 anti-human TCF1 antibody | BioLegend | 655204 |
