# Supplementary material for: Platform-based manufacturing of tumor-infiltrating lymphocytes derived from different solid tumor types
Source: Front Immunol. 2026 Oct 5;17:1854761. doi: 10.3389/fimmu.2026.1854761 (PMC13677931; doi:10.3389/fimmu.2026.1854761)
Supplement: Supplementary file 7 [file Table5.docx]

Supplementary Table 5 Process development NTU101 cell composition data throughout manufacturing

| **General batch data** | | **Sampling time** | **Cell composition** | | | | | | | | | | | |  |  |
| --- | --- | --- | --- | --- | --- | --- | --- | --- | --- | --- | --- | --- | --- | --- | --- | --- |
|  |  |  |  |  |  |  |  |  |  |  |  |  |  |  |  | |
|  |  |  | **% of viable cells** | | | | | | | | | **% of viable CD45+CD3+ cells** | | |  | |
| **Batch** | **Manufacturing site** |  | **TCRαβ+ CD8+**  **T cells** | **TCRαβ+ CD4+**  **T cells** | **TCRγδ+ T cells** | **Other CD45+CD3+** | **CD45+, CD3-, CD56+**  **(NK cells)** | **CD45+, CD14+ cells (Monocytes)** | **SSChighCD45+ CD16+ (Granulocytes)** | **CD45+, CD19+**  **(B-cells)** | **CD45- Cells** | **TCRαβ+ CD8+**  **T cells** | **TCRαβ+ CD4+**  **T cells** | **TCRαβ+ T cells** |  |  |
| ENDC01 | NKI | Tumor digest | 10.2 | 21.7 | 0.3 | 2.3 | 3.0 | 14.5 | 0.8 | 2.5 | 26.2 | 30.7 | 65.6 | 0.9 |  |  |
| ENDC02 | NKI | Tumor digest | 10.5 | 18.1 | 0.2 | 0.7 | 1.4 | 9.2 | 0.3 | 1.1 | 51.4 | 36.1 | 62.3 | 0.7 |  |  |
| ENDC03 | NKI | Tumor digest | 10.8 | 18.7 | 1.5 | 0.6 | 10.0 | 1.2 | 0.0 | 4.6 | 38.3 | 34.6 | 59.5 | 4.9 |  |  |
| ENDC04 | NKI | Tumor digest | 17.4 | 28.3 | 0.7 | 0.6 | 6.3 | 13.3 | 3.1 | 1.2 | 19.2 | 37.2 | 60.7 | 1.5 |  |  |
| ENDC05 | NKI | Tumor digest | 6.0 | 8.4 | 1.2 | 0.9 | 3.1 | 7.7 | 0.4 | 1.2 | 64.7 | 37.9 | 53.3 | 7.6 |  |  |
| ENDC06 | NKI | Tumor digest | 32.9 | 17.0 | 2.2 | 0.6 | 2.7 | 10.9 | 0.2 | 3.6 | 22.5 | 62.7 | 32.4 | 4.1 |  |  |
| ENDC07 | NKI | Tumor digest | 11.5 | 12.6 | 1.1 | 3.8 | 3.5 | 33.8 | 0.1 | 5.4 | 19.9 | 42.8 | 46.7 | 4.0 |  |  |
| ENDC08 | SBS | Tumor digest | 1.6 | 6.4 | 0.1 | 3.0 | 0.1 | 4.6 | 0.0 | 0.2 | 81.0 | 18.8 | 77.1 | 0.7 |  |  |
| ENDC09 | SBS | Tumor digest | 3.5 | 7.0 | 0.3 | 10.4 | 2.4 | 17.5 | 0.5 | 2.7 | 51.6 | 26.6 | 53.0 | 2.5 |  |  |
| NSCLC01 | NKI | Tumor digest | 4.9 | 10.6 | 0.5 | 2.7 | 0.5 | 4.7 | 0.1 | 3.4 | 70.6 | 29.8 | 63.9 | 2.8 |  |  |
| NSCLC02 | NKI | Tumor digest | 57.4 | 32.8 | 1.4 | 0.6 | 1.3 | 1.2 | 0.1 | 3.1 | 0.2 | 62.3 | 35.5 | 1.6 |  |  |
| NSCLC03 | NKI | Tumor digest | 27.5 | 19.3 | 0.7 | 1.3 | 5.1 | 30.6 | 0.6 | 3.3 | 6.8 | 57.1 | 40.0 | 1.4 |  |  |
| NSCLC04 | NKI | Tumor digest | 5.3 | 16.9 | 0.2 | 3.3 | 3.2 | 28.9 | 2.1 | 1.1 | 27.0 | 22.6 | 71.8 | 0.8 |  |  |
| NSCLC05 | NKI | Tumor digest | 4.5 | 14.8 | 0.5 | 4.1 | 2.4 | 42.8 | 1.6 | 1.0 | 21.4 | 21.4 | 70.1 | 2.4 |  |  |
| NSCLC06 | NKI | Tumor digest | 3.7 | 5.1 | 0.4 | 23.2 | 2.8 | 8.1 | 35.3 | 2.9 | 11.1 | 25.7 | 35.3 | 2.5 |  |  |
| NSCLC07 | SBS | Tumor digest | 14.9 | 25.0 | 1.0 | 14.0 | 0.7 | 8.6 | 2.7 | 4.8 | 17.5 | 29.7 | 49.8 | 2.0 |  |  |
| NSCLC08 | SBS | Tumor digest | ND | ND | ND | ND | ND | ND | ND | ND | ND | ND | ND | ND |  |  |
| NSCLC09 | SBS | Tumor digest | 10.4 | 12.5 | 1.7 | 3.4 | 0.6 | 15.4 | 0.8 | 1.1 | 10.4 | 38.5 | 46.3 | 6.5 |  |  |
| PENC01 | NKI | Tumor digest | 16.7 | 29.4 | 1.2 | 0.8 | 3.6 | 1.5 | 0.3 | 41.6 | 0.3 | 34.7 | 61.2 | 2.5 |  |  |
| PENC02 | NKI | Tumor digest | 9.5 | 13.1 | 0.4 | 0.6 | 4.9 | 33.7 | 4.0 | 4.5 | 16.9 | 40.8 | 56.4 | 1.8 |  |  |
| PENC03 | NKI | Tumor digest | 44.8 | 33.7 | 3.0 | 2.1 | 1.8 | 2.4 | 0.7 | 6.0 | 0.2 | 53.7 | 40.4 | 3.6 |  |  |
| PENC04 | NKI | Tumor digest | 51.0 | 24.4 | 0.5 | 1.3 | 3.9 | 0.0 | 2.4 | 9.3 | 0.8 | 66.2 | 31.7 | 0.6 |  |  |
| PENC05 | NKI | Tumor digest | 15.6 | 10.8 | 20.7 | 2.4 | 32.5 | 2.5 | 2.9 | 2.9 | 0.7 | 31.7 | 21.9 | 42.2 |  |  |
| PENC06 | NKI | Tumor digest | 1.7 | 29.8 | 0.2 | 1.1 | 2.6 | 6.2 | 3.4 | 46.8 | 5.5 | 5.3 | 91.3 | 0.5 |  |  |
| PENC07 | NKI | Tumor digest | 45.4 | 25.4 | 0.3 | 1.2 | 7.8 | 8.2 | 0.0 | 1.0 | 2.3 | 62.9 | 35.2 | 0.4 |  |  |
| PENC08 | NKI | Tumor digest | 2.3 | 22.0 | 0.1 | 0.7 | 2.9 | 14.6 | 0.0 | 41.1 | 4.0 | 9.1 | 88.3 | 0.5 |  |  |
| PENC09 | SBS | Tumor digest | 19.3 | 20.9 | 0.2 | 3.8 | 4.3 | 9.8 | 0.0 | 2.7 | 19.3 | 45.4 | 49.2 | 0.4 |  |  |
| PENC10 | SBS | Tumor digest | 17.6 | 45.0 | 0.3 | 4.2 | 1.5 | 3.2 | 0.0 | 0.9 | 18.3 | 26.7 | 68.1 | 0.5 |  |  |
| ENDC01 | NKI | Intermediate product | 40.4 | 31.4 | 0.0 | 4.6 | 19.9 | 0.0 | ND | 0.3 | 0.0 | 52.9 | 41.1 | 0.0 |  |  |
| ENDC02 | NKI | Intermediate product | 39.2 | 31.6 | 3.2 | 0.7 | 23.7 | 0.0 | ND | 0.0 | 0.7 | 52.5 | 42.4 | 4.3 |  |  |
| ENDC03 | NKI | Intermediate product | 12.4 | 39.8 | 8.2 | 1.0 | 32.7 | 0.0 | ND | 0.7 | 0.1 | 20.3 | 64.8 | 13.3 |  |  |
| ENDC04 | NKI | Intermediate product | 56.9 | 19.4 | 1.2 | 0.9 | 12.7 | 0.4 | ND | 0.4 | 4.9 | 72.6 | 24.7 | 1.6 |  |  |
| ENDC05 | NKI | Intermediate product | 39.3 | 28.5 | 8.8 | 1.7 | 18.9 | 0.0 | ND | 0.1 | 0.3 | 50.1 | 36.4 | 11.3 |  |  |
| ENDC06 | NKI | Intermediate product | 80.2 | 8.2 | 9.0 | 0.7 | 1.3 | 0.0 | ND | 0.0 | 0.1 | 81.7 | 8.4 | 9.2 |  |  |
| ENDC07 | NKI | Intermediate product | 35.9 | 50.5 | 0.8 | 6.4 | 4.5 | 0.0 | ND | 0.4 | 0.2 | 38.4 | 53.9 | 0.9 |  |  |
| ENDC08 | SBS | Intermediate product | 18.8 | 17.8 | 41.9 | 8.5 | 4.3 | 0.0 | ND | 0.2 | 5.7 | 21.7 | 20.6 | 48.5 |  |  |
| ENDC09 | SBS | Intermediate product | 66.1 | 11.7 | 0.3 | 2.7 | 15.9 | 0.0 | ND | 0.1 | 0.3 | 81.7 | 14.5 | 0.4 |  |  |
| NSCLC01 | NKI | Intermediate product | 53.9 | 28.1 | 1.7 | 0.6 | 13.7 | 0.0 | ND | 0.1 | 0.4 | 63.9 | 33.3 | 2.1 |  |  |
| NSCLC02 | NKI | Intermediate product | 82.4 | 7.1 | 1.1 | 0.5 | 1.0 | 0.0 | ND | 0.0 | 0.3 | 90.4 | 7.8 | 1.3 |  |  |
| NSCLC03 | NKI | Intermediate product | 42.6 | 14.6 | 5.9 | 0.3 | 32.9 | 0.0 | ND | 1.3 | 0.3 | 67.2 | 23.0 | 9.3 |  |  |
| NSCLC04 | NKI | Intermediate product | 18.6 | 57.8 | 0.8 | 5.1 | 14.5 | 0.0 | ND | 0.1 | 0.6 | 22.6 | 70.2 | 1.0 |  |  |
| NSCLC05 | NKI | Intermediate product | 73.7 | 3.5 | 9.0 | 3.0 | 10.5 | 0.0 | ND | 0.0 | 0.8 | 82.7 | 3.9 | 10.1 |  |  |
| NSCLC06 | NKI | Intermediate product | 21.0 | 6.4 | 11.4 | 3.8 | 53.2 | 0.0 | ND | 0.1 | 0.6 | 49.2 | 15.1 | 26.8 |  |  |
| NSCLC07 | SBS | Intermediate product | 33.1 | 47.0 | 2.8 | 5.8 | 8.4 | 0.0 | ND | 0.7 | 0.3 | 37.3 | 53.0 | 3.2 |  |  |
| NSCLC08 | SBS | Intermediate product | 28.1 | 58.3 | 0.3 | 10.6 | 0.6 | 0.0 | ND | 0.0 | 0.1 | 28.9 | 60.0 | 0.3 |  |  |
| NSCLC09 | SBS | Intermediate product | 8.0 | 36.9 | 5.7 | 7.4 | 30.6 | 0.0 | ND | 0.0 | 1.5 | 13.8 | 63.7 | 9.9 |  |  |
| PENC01 | NKI | Intermediate product | 83.4 | 11.3 | 3.3 | 1.1 | 0.8 | 0.0 | ND | 0.0 | 0.0 | 84.1 | 11.4 | 3.3 |  |  |
| PENC02 | NKI | Intermediate product | 62.9 | 23.4 | 1.3 | 1.0 | 9.6 | 0.0 | ND | 0.0 | 0.0 | 71.0 | 26.4 | 1.5 |  |  |
| PENC03 | NKI | Intermediate product | 77.9 | 3.1 | 15.1 | 1.3 | 4.7 | 0.0 | ND | 0.0 | 0.0 | 80.0 | 3.2 | 15.5 |  |  |
| PENC04 | NKI | Intermediate product | 71.1 | 18.0 | 1.6 | 0.7 | 7.5 | 0.0 | ND | 0.1 | 0.0 | 77.8 | 19.7 | 1.8 |  |  |
| PENC05 | NKI | Intermediate product | 23.5 | 5.8 | 30.6 | 0.4 | 38.4 | 0.0 | ND | 0.0 | 0.1 | 38.9 | 9.6 | 50.8 |  |  |
| PENC06 | NKI | Intermediate product | 11.6 | 63.1 | 0.5 | 1.9 | 18.9 | 0.0 | ND | 1.0 | 0.8 | 15.0 | 81.9 | 0.6 |  |  |
| PENC07 | NKI | Intermediate product | 68.0 | 5.1 | 1.9 | 0.8 | 23.8 | 0.0 | ND | 0.0 | 0.0 | 89.6 | 6.8 | 2.6 |  |  |
| PENC08 | NKI | Intermediate product | 14.6 | 42.6 | 1.2 | 7.4 | 24.6 | 0.0 | ND | 6.2 | 0.1 | 22.2 | 64.7 | 1.9 |  |  |
| PENC09 | SBS | Intermediate product | 60.0 | 0.4 | 2.1 | 8.6 | 17.7 | 0.0 | ND | 0.0 | 0.4 | 84.3 | 0.6 | 2.9 |  |  |
| PENC10 | SBS | Intermediate product | 25.8 | 18.9 | 0.5 | 4.6 | 37.5 | 0.0 | ND | 0.5 | 10.6 | 52.3 | 38.4 | 1.0 |  |  |
| ENDC01 | NKI | NTU101 drug product | 48.0 | 49.1 | 0.1 | 1.1 | 0.5 | 0.0 | ND | 0.0 | 0.1 | 48.9 | 50.0 | 0.1 |  |  |
| ENDC02 | NKI | NTU101 drug product | 45.1 | 50.9 | 0.8 | 1.7 | 0.2 | 0.1 | ND | 0.0 | 0.2 | 45.8 | 51.7 | 0.8 |  |  |
| ENDC03 | NKI | NTU101 drug product | 51.0 | 34.8 | 8.0 | 1.5 | 2.9 | 0.0 | ND | 0.0 | 0.0 | 53.5 | 36.5 | 8.4 |  |  |
| ENDC04 | NKI | NTU101 drug product | 67.0 | 29.3 | 0.2 | 1.9 | 0.2 | 0.0 | ND | 0.1 | 0.1 | 68.0 | 29.8 | 0.2 |  |  |
| ENDC05 | NKI | NTU101 drug product | 75.6 | 19.0 | 2.9 | 1.2 | 0.2 | 0.0 | ND | 0.0 | 0.2 | 76.6 | 19.2 | 2.9 |  |  |
| ENDC06 | NKI | NTU101 drug product | 83.9 | 6.9 | 8.1 | 0.9 | 0.2 | 0.0 | ND | 0.0 | 0.1 | 84.1 | 6.9 | 8.1 |  |  |
| ENDC07 | NKI | NTU101 drug product | 6.7 | 88.1 | 0.0 | 0.3 | 0.6 | 0.0 | ND | 0.0 | 0.1 | 7.0 | 92.6 | 0.0 |  |  |
| ENDC08 | SBS | NTU101 drug product | 22.7 | 64.7 | 8.9 | 2.5 | 0.7 | 0.0 | ND | 0.0 | 0.1 | 23.0 | 65.5 | 9.0 |  |  |
| ENDC09 | SBS | NTU101 drug product | 80.2 | 15.9 | 0.6 | 1.0 | 3.6 | 0.0 | ND | 0.0 | 0.1 | 82.0 | 16.3 | 0.7 |  |  |
| NSCLC01 | NKI | NTU101 drug product | 61.2 | 37.0 | 0.2 | 1.4 | 0.8 | 0.0 | ND | 0.0 | 0.0 | 61.3 | 37.1 | 0.2 |  |  |
| NSCLC02 | NKI | NTU101 drug product | 90.0 | 7.4 | 1.0 | 0.5 | 0.0 | 0.0 | ND | 0.0 | 0.2 | 91.0 | 7.5 | 1.0 |  |  |
| NSCLC03 | NKI | NTU101 drug product | 74.8 | 22.3 | 0.4 | 1.3 | 0.2 | 0.0 | ND | 0.1 | 0.1 | 75.8 | 22.6 | 0.4 |  |  |
| NSCLC04 | NKI | NTU101 drug product | 9.9 | 87.5 | 0.1 | 1.4 | 0.1 | 0.0 | ND | 0.0 | 0.5 | 10.0 | 88.4 | 0.1 |  |  |
| NSCLC05 | NKI | NTU101 drug product | 51.3 | 45.1 | 1.2 | 1.3 | 0.3 | 0.0 | ND | 0.0 | 0.2 | 51.9 | 45.6 | 1.2 |  |  |
| NSCLC06 | NKI | NTU101 drug product | 61.0 | 28.9 | 7.5 | 1.7 | 0.3 | 0.0 | ND | 0.2 | 0.0 | 61.5 | 29.2 | 7.6 |  |  |
| NSCLC07 | SBS | NTU101 drug product | 49.3 | 40.4 | 0.9 | 7.8 | 0.2 | 0.0 | ND | 0.0 | 0.3 | 50.1 | 41.1 | 0.9 |  |  |
| NSCLC08 | SBS | NTU101 drug product | 28.8 | 68.5 | 0.2 | 2.3 | 0.0 | 0.0 | ND | 0.0 | 0.0 | 28.9 | 68.7 | 0.2 |  |  |
| NSCLC09 | SBS | NTU101 drug product | 20.8 | 69.9 | 0.4 | 7.0 | 0.1 | 0.0 | ND | 0.0 | 0.2 | 21.2 | 71.3 | 0.5 |  |  |
| PENC01 | NKI | NTU101 drug product | 93.0 | 5.7 | 0.7 | 0.4 | 0.0 | 0.0 | ND | 0.0 | 0.0 | 93.1 | 5.7 | 0.7 |  |  |
| PENC02 | NKI | NTU101 drug product | 75.7 | 20.4 | 0.6 | 1.4 | 0.4 | 0.0 | ND | 0.0 | 0.2 | 77.1 | 20.8 | 0.6 |  |  |
| PENC03 | NKI | NTU101 drug product | 81.4 | 5.6 | 11.5 | 0.8 | 0.5 | 0.0 | ND | 0.0 | 0.0 | 81.9 | 5.7 | 11.6 |  |  |
| PENC04 | NKI | NTU101 drug product | 92.7 | 5.7 | 0.4 | 0.6 | 0.1 | 0.0 | ND | 0.0 | 0.0 | 93.2 | 5.7 | 0.4 |  |  |
| PENC05 | NKI | NTU101 drug product | 73.0 | 15.4 | 5.9 | 0.8 | 4.1 | 0.0 | ND | 0.0 | 0.2 | 76.8 | 16.2 | 6.2 |  |  |
| PENC06 | NKI | NTU101 drug product | 27.5 | 67.5 | 0.7 | 1.7 | 0.2 | 0.0 | ND | 0.0 | 0.5 | 28.2 | 69.3 | 0.7 |  |  |
| PENC07 | NKI | NTU101 drug product | 89.3 | 5.0 | 0.1 | 3.4 | 0.1 | 0.0 | ND | 0.0 | 0.0 | 91.3 | 5.1 | 0.1 |  |  |
| PENC08 | NKI | NTU101 drug product | 14.3 | 77.9 | 0.7 | 5.0 | 1.1 | 0.0 | ND | 0.3 | 0.0 | 14.6 | 79.6 | 0.7 |  |  |
| PENC09 | SBS | NTU101 drug product | 96.1 | 0.8 | 0.1 | 1.3 | 1.1 | 0.0 | ND | 0.1 | 0.2 | 97.7 | 0.8 | 0.1 |  |  |
| PENC10 | SBS | NTU101 drug product | 84.4 | 12.2 | 0.0 | 2.7 | 0.3 | 0.0 | ND | 0.0 | 0.2 | 85.0 | 12.3 | 0.0 |  |  |

*ENDC = Endometrial carcinoma, NSCLC = Non-small cell lung cancer, PENC = Penile cancer, NKI = Netherlands Cancer Institute, SBS = Sanquin Blood Supply, ND = no data*
