# Supplementary material for: Platform-based manufacturing of tumor-infiltrating lymphocytes derived from different solid tumor types
Source: Front Immunol. 2026 Oct 5;17:1854761. doi: 10.3389/fimmu.2026.1854761 (PMC13677931; doi:10.3389/fimmu.2026.1854761)
Supplement: Supplementary file 8 [file Table6.docx]

Supplementary Table 6 Process development NTU101 manufacturing and in-process control (IPC) data

| **General batch data** | | **Step 2:**  **Cell isolation** | | **Step 3a: Proliferation** | | | | | | | | **Steps 4 and 5: Rapid Expansion Phase (REP)** | | | | | | | | | | |
| --- | --- | --- | --- | --- | --- | --- | --- | --- | --- | --- | --- | --- | --- | --- | --- | --- | --- | --- | --- | --- | --- | --- |
|  |  |  |  | **Start** | | **End** | | | | | | **Start** | | | | **Day 6** | | | **End** | | | |
| **Batch** | **Manufacturing site** | **Total tumor volume (cm^3^)** | **% Viable CD45+CD3+ cells** | **Viable cell number (×10^6^)** | **Viable CD3+CD45+ cell number (×10^6^)** | **Viable cell number (×10^6^)** | **% Viable CD45+CD3+ cells** | **Viable CD45+CD3+ cell number (×10^6^)** | **Viable non-tumor cell fold expansion (x)** | **Viable CD45+CD3+ cell fold expansion** | **% Viable tumor cell** | **Viable cell number (×10^6^)** | **Viable CD45+CD3+ cell number (×10^6^)** | **Feeder : viable CD45+CD3+ cell ratio** | **Viable cell fold expansion** | | **Feeder cell – proliferation** | **Viable cell number (×10^6^)** | **Viable cell number (×10^9^)** | **Viable cell fold expansion** | **Viable CD45+CD3+ cell number (×10^9^)** | **Viable CD45+CD3+ cell fold expansion** |
| **limit** |  |  |  |  |  |  | IPC2: ≥ 10.0 % | IPC3: ≥ 10×10^6^ cells | IPC4: > 2-fold |  | ICP5: < 10 % |  | IPC7: ≥ 0.5×10^6^ cells | IPC8: 100-200:1 | IPC9: > 10-fold | | IPC10: < 1.0-fold | IPC11: NA |  |  |  |  |
| ENDC01 | NKI | 3.00 | 34.5 | 23.2 | 8.0 | 226.8 | 76.4 | 173.3 | 40 | 21.6 | 0 | 1.0 | 0.8 | 222 | 128 | | 0.2 | 97.5 | 1.6 | 1572 | 1.12 | 1466 |
| ENDC02 | NKI | 22.50 | 29.5 | 12.5 | 3.7 | 140.4 | 74.7 | 104.9 | 37 | 28.4 | 0 | 1.0 | 0.7 | 228 | 60 | | 0.2 | 45.0 | 1.3 | 1342 | 1.43 | 1908 |
| ENDC03 | NKI | 1.80 | 31.7 | 19.8 | 6.3 | 87.7 | 61.3 | 53.8 | 16 | 8.6 | 0 | 1.9 | 1.1 | 148 | 35 | | 0.2 | 40.5 | 1.3 | 720 | 1.18 | 1031 |
| ENDC04 | NKI | 1.00 | 47.0 | 6.2 | 2.9 | 120.7 | 78.4 | 94.6 | 62 | 32.7 | 0 | 1.0 | 0.8 | 182 | 97 | | 0.4 | 76.3 | 1.8 | 1798 | 1.35 | 1725 |
| ENDC05 | NKI | 0.60 | 16.5 | 2.0 | 0.3 | 15.7 | 78.3 | 12.3 | 31 | 36.7 | 0 | 1.0 | 0.8 | 186 | 95 | | 0.4 | 73.0 | 1.0 | 1056 | 0.86 | 1114 |
| ENDC06 | NKI | 36.00 | 52.7 | 10.0 | 5.3 | 111.7 | 98.1 | 109.5 | 26 | 20.7 | 0 | 1.0 | 1.0 | 108 | 86 | | 0.5 | 84.3 | 1.5 | 1519 | 1.06 | 1077 |
| ENDC07 | NKI | ND | 29.0 | 0.5 | 0.1 | 9.8 | 93.6 | **9.1** | 46 | 63.1 | 0 | 0.8 | 0.8 | 226 | 47 | | 0.1 | 36.8 | 0.7 | 823 | 0.52 | 668 |
| ENDC08 | SBS | ND | 11.0 | 27.7 | 3.0 | 36.8 | 87.1 | 32.0 | 7 | 10.5 | 1 | 1.0 | 0.9 | 230 | 57 | | **2.3^1^** | 49.3 | 1.3 | 1315 | 1.66 | 1902 |
| ENDC09 | SBS | 15.60 | 21.3 | 6.4 | 1.4 | 26.6 | 80.9 | 21.5 | 38 | 15.9 | 0 | 1.0 | 0.8 | 247 | 48 | | 0.1 | 39.0 | 0.8 | 845 | 0.83 | 1021 |
| NSCLC01 | NKI | 4.50 | 18.7 | 8.4 | 1.6 | 60.9 | 84.4 | 51.4 | 20 | 32.6 | 0 | 1.0 | 0.8 | 115 | 45 | | 0.5 | 38.0 | 1.0 | 983 | 1.12 | 1323 |
| NSCLC02 | NKI | 0.13 | 92.1 | 1.5 | 1.4 | 106.3 | 91.1 | 96.9 | 78 | 68.7 | 0 | 1.0 | 0.9 | 107 | 46 | | 0.5 | 41.5 | 1.8 | 1848 | 0.92 | 1013 |
| NSCLC03 | NKI | 10.00 | 48.8 | 1.6 | 0.8 | 55.6 | 63.5 | 35.3 | 59 | 44.5 | 0 | 1.0 | 0.6 | 153 | 60 | | 0.5 | 37.8 | 0.9 | 900 | 0.89 | 1401 |
| NSCLC04 | NKI | 6.00 | 25.7 | 5.8 | 1.5 | 82.1 | 82.4 | 67.6 | 39 | 45.3 | 0 | 1.0 | 0.8 | 164 | 59 | | **1.1^1^** | 48.8 | 1.0 | 1035 | 0.99 | 1205 |
| NSCLC05 | NKI | 18.00 | 23.9 | 3.4 | 0.8 | 38.5 | 89.2 | 34.3 | 36 | 41.8 | 0 | 1.0 | 0.9 | 158 | 55 | | 0.8 | 49.8 | 1.3 | 1322 | 1.29 | 1419 |
| NSCLC06 | NKI | 8.21 | 32.3 | 4.1 | 1.3 | 19.4 | 42.7 | **8.3** | 7 | 6.3 | 0 | 1.5 | 0.6 | 318 | 34 | | 0.2 | 21.5 | 0.6 | 429 | 0.66 | 1051 |
| NSCLC07 | SBS | 4.50 | 55.0 | 46.0 | 25.3 | 185.6 | 88.7 | 164.6 | 11 | 6.5 | 5 | 1.0 | 0.9 | 225 | 40 | | 0.2 | 35.8 | 1.3 | 1330 | 1.09 | 1230 |
| NSCLC08 | SBS | ND | ND | 0.9 | ND | 197.2 | 97.3 | 191.9 | 408 | ND | 9 | 1.8 | 1.7 | 116 | 39 | | **2.5^1^** | 67.8 | 1.5 | 835 | 1.55 | 899 |
| NSCLC09 | SBS | ND | 28.0 | 0.3 | 0.1 | 4.3 | 58.0 | **2.5** | 144 | 33.8 | 0 | 1.0 | 0.6 | 345 | 86 | | 0.4 | 49.8 | 1.0 | 1042 | 0.79 | 1363 |
| PENC01 | NKI | 3.00 | 48.1 | 1.5 | 0.7 | 12.9 | 99.1 | 12.8 | 10 | 18.2 | 0 | 0.9 | 0.9 | 140 | 57 | | 0.2 | 52.5 | 1.8 | 1925 | 1.32 | 1428 |
| PENC02 | NKI | 4.50 | 23.6 | 5.0 | 1.2 | 46.7 | 88.6 | 41.4 | 62 | 35.4 | 0 | 1.0 | 0.9 | 147 | 69 | | 0.2 | 60.8 | 1.9 | 1887 | 1.04 | 1168 |
| PENC03 | NKI | 1.00 | 83.5 | 1.4 | 1.2 | 135.9 | 97.4 | 132.3 | 162 | 110.1 | 0 | 1.0 | 1.0 | 132 | 46 | | 0.2 | 45.0 | 1.7 | 1710 | 1.49 | 1510 |
| PENC04 | NKI | 1.20 | 77.1 | 24.9 | 19.2 | 129.7 | 91.5 | 118.6 | 16 | 6.2 | 0 | 1.0 | 0.9 | 139 | 85 | | 0.2 | 79.5 | 2.2 | 2104 | 1.48 | 1581 |
| PENC05 | NKI | 25.00 | 49.4 | 2.6 | 1.3 | 17.5 | 60.3 | 10.5 | 76 | 8.2 | 0 | 1.2 | 0.7 | 152 | 77 | | 0.5 | 53.5 | 1.2 | 993 | 0.72 | 1028 |
| PENC06 | NKI | 8.00 | 32.8 | 2.0 | 0.7 | 21.0 | 77.0 | 16.2 | 12 | 24.7 | 0 | 1.0 | 0.7 | 199 | 19 | | 0.0 | 14.0 | 1.7 | 1725 | 1.33 | 1803 |
| PENC07 | NKI | 2.00 | 72.3 | 2.0 | 1.5 | 71.6 | 75.9 | 54.3 | 171 | 37.3 | 0 | 1.0 | 0.8 | 200 | 57 | | 0.5 | 43.1 | 1.3 | 1277 | 0.89 | 1186 |
| PENC08 | NKI | 1.125 | 25.3 | 2.0 | 0.5 | 25.8 | 65.8 | 17.0 | 15 | 33.8 | 0 | 0.9 | 0.6 | 197 | 55 | | 0.2 | 33.8 | 0.6 | 666 | 0.47 | 758 |
| PENC09 | SBS | 0.88 | 44.2 | 4.0 | 1.8 | 51.8 | 71.1 | 36.9 | 14 | 21.0 | 0 | 0.8 | 0.5 | 375 | 213 | | 0.1 | 113.5 | 1.6 | 2125 | 1.57 | 2939 |
| PENC10 | SBS | 0.38 | 67.2 | 0.03 | 0.02 | 0.43 | 49.8 | **0.21** | 14 | 10.6 | 6 | 0.2 | **0.1** | ND | 378 | | ND | 43.3 | 1.1 | 4925 | 1.12 | 9812 |

*Bold = parameters that did not meet IPC action limits; ENDC = Endometrial carcinoma, NSCLC = Non-small cell lung cancer, PENC = Penile cancer, NKI= Netherlands Cancer Institute, SBS = Sanquin Blood Supply, ND = no data, 1= The observed feeder cell ‘proliferation’ of >1 fold was considered an artifact due to the (im-)precision of the counting performed after thawing of the feeder cells and at day 6 of the REP, and not due to resistance to the used irradiation dose of PBMCs, as the feeder cell morphologies in the brightfield microscope were consistent with successful irradiation.*
