# Supplementary material for: Platform-based manufacturing of tumor-infiltrating lymphocytes derived from different solid tumor types
Source: Front Immunol. 2026 Oct 5;17:1854761. doi: 10.3389/fimmu.2026.1854761 (PMC13677931; doi:10.3389/fimmu.2026.1854761)
Supplement: Supplementary file 9 [file Table7.docx]

Supplementary Table 7 Process development NTU101 Regulatory T cell (T-reg) composition data in the tumor digest

| **General batch data** | | **T-reg composition** | |  |
| --- | --- | --- | --- | --- |
|  |  |  |  |  |
|  |  | **% of CD4+ T cells** | |  |
| **Batch** | **Manufacturing site** | **CD25+CD127_low_ FoxP3+ (T-regs)** | **CD25-CD127_high_ FoxP3- (Conventional CD4 T cells)** |  |
| ENDC01 | NKI | 0.0 | 100.0 |  |
| ENDC02 | NKI | 0.0 | 100.0 |  |
| ENDC03 | NKI | 28.9 | 71.1 |  |
| ENDC04 | NKI | 18.2 | 81.8 |  |
| ENDC05 | NKI | 8.2 | 91.8 |  |
| ENDC06 | NKI | 0.3 | 99.7 |  |
| ENDC07 | NKI | ND | ND |  |
| ENDC08 | SBS | 0.3 | 99.7 |  |
| ENDC09 | SBS | 22.8 | 77.2 |  |
| NSCLC01 | NKI | 7.1 | 92.9 |  |
| NSCLC02 | NKI | 18.9 | 81.1 |  |
| NSCLC03 | NKI | 25.5 | 74.5 |  |
| NSCLC04 | NKI | ND | ND |  |
| NSCLC05 | NKI | ND | ND |  |
| NSCLC06 | NKI | 22.1 | 77.9 |  |
| NSCLC07 | SBS | 1.0 | 99.0 |  |
| NSCLC08 | SBS | ND | ND |  |
| NSCLC09 | SBS | 3.2 | 96.8 |  |
| PENC01 | NKI | 18.9 | 81.1 |  |
| PENC02 | NKI | 17.2 | 82.8 |  |
| PENC03 | NKI | ND | ND |  |
| PENC04 | NKI | 18.8 | 81.2 |  |
| PENC05 | NKI | 7.1 | 92.9 |  |
| PENC06 | NKI | ND | ND |  |
| PENC07 | NKI | 9.7 | 90.3 |  |
| PENC08 | NKI | 44.6 | 55.4 |  |
| PENC09 | SBS | 14.4 | 85.6 |  |
| PENC10 | SBS | 12.4 | 87.6 |  |

*ENDC = Endometrial carcinoma, NSCLC = Non-small cell lung cancer, PENC = Penile cancer, NKI = Netherlands Cancer Institute, SBS = Sanquin Blood Supply, ND = no data*
