# Supplementary material for: Platform-based manufacturing of tumor-infiltrating lymphocytes derived from different solid tumor types
Source: Front Immunol. 2026 Oct 5;17:1854761. doi: 10.3389/fimmu.2026.1854761 (PMC13677931; doi:10.3389/fimmu.2026.1854761)
Supplement: Supplementary file 10 [file Table8.docx]

Supplementary Table 8 Process development NTU101 drug product (DP) Release Test (RT) data

| **General batch data** | | **PD NTU101 DP** | | | | | |
| --- | --- | --- | --- | --- | --- | --- | --- |
| **Batch** | **Manufacturing site** | **IFN-γ release (pg/mL) (×10^3^)** | **Viable CD45+CD3+ cells (%)** | **Viable CD45- cells (%)** | **Viable CD19+ cells (%)** | **Viability (%)** | **Viable CD45+CD3+ cell number (×10^9^)**  **(scale-compensated)** |
| **limit** |  | RT1: ≥ 1.0×10^3^ pg/mL | RT3: ≥ 90.0 % | RT4: < 1% | RT5: < 1% | RT8: ≥ 70 % | RT9: ≥ 10×10^9^ cells |
| ENDC01 | NKI | 2.5 | 98.2 | <LOD | <LOD | 97.8 | 22.4 |
| ENDC02 | NKI | 2.9 | 98.5 | <LOD | <LOD | 97.6 | 28.5 |
| ENDC03 | NKI | 5.5 | 95.2 | <LOD | <LOD | 97.5 | 23.6 |
| ENDC04 | NKI | 1.7 | 98.5 | <LOD | <LOD | 97.9 | 27.1 |
| ENDC05 | NKI | 1.2 | 98.6 | <LLOQ | <LOD | 96.0 | 17.1 |
| ENDC06 | NKI | 1.7 | 99.7 | <LOD | <LOD | 94.9 | 21.1 |
| ENDC07 | NKI | 2.0 | 95.1 | <LOD | <LOD | 98.2 | 10.5^2^ |
| ENDC08 | SBS | 4.5 | 98.8 | <LLOQ | <LOD | 99.9 | 33.1 |
| ENDC09 | SBS | 4.8 | 97.8 | <LLOQ | <LOD | 98.0 | 16.2 |
| NSCLC01 | NKI | 4.2 | 99.8 | <LOD | <LOD | 95.4 | 22.3 |
| NSCLC02 | NKI | 4.2 | 98.9 | <LOD | <LOD | 97.5 | 18.5 |
| NSCLC03 | NKI | 4.2 | 98.8 | <LOD | <LOD | 98.0 | 17.8 |
| NSCLC04 | NKI | **0.8** | 99.0 | <LLOQ | <LOD | 97.6 | 19.9 |
| NSCLC05 | NKI | 1.7 | 99.0 | <LOD | <LOD | 97.4 | 25.8 |
| NSCLC06 | NKI | 2.6 | 99.1 | <LOD | <LLOQ | 95.7 | 13.2^2^ |
| NSCLC07 | SBS | 3.3 | 98.3 | <LLOQ | <LOD | 98.7 | 21.8 |
| NSCLC08 | SBS | **0.8** | 99.8 | <LOD | <LOD | 99.6 | 31.0 |
| NSCLC09 | SBS | 4.8 | 98.1 | <LLOQ | <LOD | 99.8 | 16.1^2^ |
| PENC01 | NKI | 2.6 | 99.9 | <LOD | <LOD | 97.6 | 26.5 |
| PENC02 | NKI | 1.0 | 98.2 | <LOD | <LOD | 98.4 | 20.7 |
| PENC03 | NKI | 4.0 | 99.5 | <LOD | <LOD | 97.8 | 29.8 |
| PENC04 | NKI | 2.1 | 99.4 | <LOD | <LOD | 98.3 | 29.6 |
| PENC05 | NKI | 2.5 | 95.1 | <LLOQ | <LOD | 95.3 | 14.4 |
| PENC06 | NKI | 1.4 | 97.4 | <LLOQ | <LOD | 96.2 | 26.7 |
| PENC07 | NKI | 1.9 | 97.9 | <LOD | <LOD | 93.8 | 17.8 |
| PENC08 | NKI | 2.1 | 97.8 | <LOD | <LLOQ | 99.6 | **9.4^1^** |
| PENC09 | SBS | 4.5 | 98.4 | <LLOQ | <LOD | 99.0 | 31.9 |
| PENC10 | SBS | 4.1 | 100.0 | <LOD | <LOD | 99.3 | 22.7^2^ |

*1 = PENC8 had 17×10^6 viable CD45+CD3+ cells available in the NTU101 intermediate product, but the REP was started with 0.6×10^6 viable CD45+CD3+ cells. Based on the observed expansion of CD45+CD3+ cells (995-fold) during the REP, PEN8 would have met the 10×10^9 T cell dose target if it had started with all available viable CD45+CD3+ cells in the end-of-proliferation NTU101 and could thus be counted as a success.*

*2 = A full-scale REP, started with 20×10^6 viable CD45+CD3+ cells, would not have been possible for these batches, so the 20-fold multiplication used to calculate these cell numbers is not applicable. However, it was still used, as the starting material dimensions will be larger during GMP NTU101 manufacturing , likely resulting in more CD45+CD3+ cells in the NTU101 intermediate product, and when expanded using a Xuri W25 Bioreactor instead of in T-175 flasks in the REP, viable CD45+CD3+ cells would have expanded more.*

*Bold = parameters that did not meet RT acceptance criteria; ENDC = Endometrial carcinoma, NSCLC = Non-small cell lung cancer, PENC = Penile cancer, NKI = Netherlands Cancer Institute, SBS = Sanquin Blood Supply, LLOQ = lower limit of quantification (<1%), LOD = limit of detection (<0.2%)*
