# Supplementary material for: Platform-based manufacturing of tumor-infiltrating lymphocytes derived from different solid tumor types
Source: Front Immunol. 2026 Oct 5;17:1854761. doi: 10.3389/fimmu.2026.1854761 (PMC13677931; doi:10.3389/fimmu.2026.1854761)
Supplement: Supplementary file 11 [file Table9.docx]

Supplementary Table 9 Process development NTU101 drug product (DP) T-cell differentiation data

| **General batch data** | | **T-cell differentiation** | | | | | | | | | |  |  |
| --- | --- | --- | --- | --- | --- | --- | --- | --- | --- | --- | --- | --- | --- |
|  |  | **% of CD8 T cells** | | | | | **% of CD4 T cells** | | | | |  | |
| **Batch** | **Manufacturing site** | **CD27- CD45RA+ CD95- cells (EMRA)** | **CD27- CD45RA+ CD95+ cells (EFF)** | **CD27+/- CD45RA- CCR7- cells (EM)** | **CD27+/-CD45RA-CCR7+ cells (CM)** | **CD27+ CD45RA+ cells (Naive-like)** | **CD27- CD45RA+ CD95- cells (EMRA)** | **CD27- CD45RA+ CD95+ cells (EFF)** | **CD27+/- CD45RA- CCR7- cells (EM)** | **CD27+/-CD45RA-CCR7+ cells (CM)** | **CD27+ CD45RA+ cells (Naive-like)** |  |  |
| ENDC01 | NKI | 0.0% | 0.1% | 90.0% | 9.9% | 0.0% | 0.0% | 0.2% | 92.9% | 6.9% | 0.0% |  |  |
| ENDC02 | NKI | 0.0% | 0.0% | 85.5% | 14.5% | 0.0% | 0.0% | 0.2% | 94.1% | 5.7% | 0.0% |  |  |
| ENDC03 | NKI | 0.0% | 0.0% | 87.4% | 12.6% | 0.0% | 0.0% | 0.0% | 94.1% | 5.8% | 0.0% |  |  |
| ENDC04 | NKI | 0.0% | 0.0% | 98.9% | 1.0% | 0.0% | 0.0% | 0.1% | 98.7% | 1.2% | 0.0% |  |  |
| ENDC05 | NKI | 0.0% | 0.1% | 92.9% | 6.9% | 0.0% | 0.0% | 0.1% | 94.6% | 5.2% | 0.0% |  |  |
| ENDC06 | NKI | 0.0% | 0.7% | 98.0% | 1.2% | 0.0% | 0.0% | 0.2% | 99.0% | 0.5% | 0.3% |  |  |
| ENDC07 | NKI | 0.0% | 0.5% | 93.8% | 3.5% | 0.4% | 0.0% | 0.1% | 98.7% | 1.0% | 0.1% |  |  |
| ENDC08 | SBS | 0.0% | 0.0% | 83.9% | 15.1% | 0.0% | 0.0% | 0.0% | 98.7% | 0.7% | 0.0% |  |  |
| ENDC09 | SBS | 0.0% | 0.2% | 95.0% | 4.7% | 0.1% | 0.0% | 0.6% | 91.6% | 7.3% | 0.5% |  |  |
| NSCLC01 | NKI | 0.0% | 0.0% | 59.5% | 40.5% | 0.0% | 0.0% | 1.6% | 92.0% | 6.4% | 0.0% |  |  |
| NSCLC02 | NKI | 0.0% | 0.0% | 85.7% | 14.3% | 0.0% | 0.0% | 0.4% | 92.1% | 7.5% | 0.1% |  |  |
| NSCLC03 | NKI | 0.0% | 0.9% | 96.0% | 2.5% | 0.0% | 0.0% | 0.1% | 98.2% | 0.7% | 0.1% |  |  |
| NSCLC04 | NKI | 0.0% | 0.0% | 95.8% | 4.1% | 0.0% | 0.0% | 0.2% | 99.0% | 0.7% | 0.0% |  |  |
| NSCLC05 | NKI | 0.0% | 0.7% | 75.7% | 23.2% | 0.1% | 0.0% | 0.4% | 97.0% | 2.3% | 0.0% |  |  |
| NSCLC06 | NKI | 0.0% | 1.1% | 86.8% | 11.7% | 0.1% | 0.0% | 1.2% | 95.8% | 2.7% | 0.1% |  |  |
| NSCLC07 | SBS | 0.0% | 0.2% | 82.2% | 17.5% | 0.0% | 0.0% | 0.4% | 96.6% | 2.7% | 0.0% |  |  |
| NSCLC08 | SBS | 0.0% | 0.0% | 82.5% | 17.2% | 0.0% | 0.0% | 0.0% | 96.0% | 4.0% | 0.0% |  |  |
| NSCLC09 | SBS | 0.0% | 0.0% | 40.9% | 59.1% | 0.0% | 0.0% | 0.6% | 90.1% | 8.7% | 0.1% |  |  |
| PENC01 | NKI | 0.0% | 0.7% | 98.8% | 0.5% | 0.1% | 0.0% | 0.5% | 96.4% | 2.9% | 0.1% |  |  |
| PENC02 | NKI | 0.0% | 0.9% | 98.1% | 0.9% | 0.0% | 0.0% | 0.1% | 88.2% | 11.8% | 0.0% |  |  |
| PENC03 | NKI | 0.0% | 2.6% | 97.1% | 0.2% | 0.0% | 0.0% | 0.2% | 87.9% | 11.9% | 0.0% |  |  |
| PENC04 | NKI | 0.0% | 0.1% | 99.6% | 0.3% | 0.0% | 0.0% | 0.0% | 87.1% | 12.9% | 0.0% |  |  |
| PENC05 | NKI | 0.0% | 0.1% | 99.1% | 0.8% | 0.0% | 0.0% | 0.1% | 83.8% | 16.0% | 0.1% |  |  |
| PENC06 | NKI | 0.0% | 0.7% | 97.2% | 2.0% | 0.0% | 0.0% | 0.0% | 84.9% | 15.1% | 0.0% |  |  |
| PENC07 | NKI | 0.0% | 0.0% | 99.8% | 0.2% | 0.0% | 0.0% | 0.2% | 88.2% | 11.6% | 0.0% |  |  |
| PENC08 | NKI | 0.0% | 0.1% | 93.0% | 4.5% | 1.2% | 0.0% | 0.1% | 72.6% | 26.4% | 0.4% |  |  |
| PENC09 | SBS | 0.0% | 0.1% | 95.5% | 4.3% | 0.0% | 0.0% | 4.2% | 74.4% | 16.5% | 5.0% |  |  |
| PENC10 | SBS | 0.0% | 3.3% | 87.1% | 8.9% | 0.6% | 0.0% | 1.0% | 77.9% | 21.0% | 0.2% |  |  |

*ENDC = Endometrial carcinoma, NSCLC = Non-small cell lung cancer, PENC = Penile cancer, NKI= Netherlands Cancer Institute, SBS = Sanquin Blood Supply, ND = no data*
